# Switching on Versatility: Recent Advances in Switchable Plasmonic Nanostructures

**DOI:** 10.1002/smsc.202300048

**Published:** 2023-09-10

**Authors:** Hajun Yoo, Hyunwoong Lee, Seongmin Im, Sukhyeon Ka, Gwiyeong Moon, Kyungnam Kang, Donghyun Kim

**Affiliations:** ^1^ School of Electrical and Electronic Engineering Yonsei University Seoul 03722 Korea

**Keywords:** active plasmonics, dynamic optical devices, energy harvesting, nanophotonics, plasmonic nanostructures, surface plasmon resonances, switchable nanostructures

## Abstract

Plasmonic nanostructures are emerging as a promising avenue for nanophotonics due to their extreme light and thermal confinement, ultrafast manipulation processes, and potential uses in device miniaturization. However, their fixed functions have limited their versatility in applications. This review provides an overview of recent switchable plasmonic nanostructure engineering techniques, focusing on methods that provide reversible switchability. Passive optical switching, active structure‐tunable switching, active material‐based switching, and advanced applications, such as multifunctional biomedical sensing, energy harvesting, and dynamic optical devices, are discussed. The specific methods and techniques used to engineer switchable plasmonic nanostructures are also highlighted. By understanding the latest developments and overall trends, this review is expected to help researchers design and fabricate advanced plasmonic nanostructures with unprecedented switchability and versatility for various applications.

## Graphical abstract

Recent advances in switchable plasmonic nanostructures, which can be reversibly switched between different configurations or functionalities, are reviewed, wherein three types of switching mechanisms are covered, namely, passive optical switching, active structure‐tunable switching, and active material‐based switching. Their applications in biomedical sensing, energy harvesting, and dynamic optical devices are also discussed.

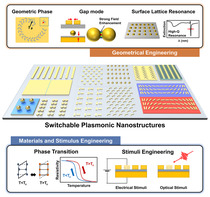

## 1 Introduction

Plasmonics is a promising field of research that deals with the interaction between light and metallic structures at nanoscale dimensions. An important aspect of plasmonics is the excitation of surface plasmon polaritons (SPPs), representing light coupling with the collective oscillation of free electrons at the metal/dielectric interface.^[^ 1, 2 ^]^ When light interacts with a thin metal surface, SPP excitation occurs along the surface, resulting in a strongly localized electromagnetic field at the interface. SPPs are typically associated with propagating waves that extend across a metal–dielectric interface, leading to vast advances in applications such as surface plasmon resonance (SPR) sensing and imaging.^[^ 3, 4, 5, 6 ^]^ Another important aspect of plasmonics is localized SPR (LSPR). LSPR occurs when the collective electron oscillation is confined to a subwavelength region or a nanoscale structure itself, leading to highly enhanced electromagnetic local fields.^[^ 1, 2, 7 ^]^ LSPR is advantageous because of its extreme light and heat confinement, high‐speed manipulation processes, and potential for device miniaturization.^[^ ^8^ ^]^ In this regard, plasmonics have fueled diverse applications, including ultrasensitive biosensing,^[^ 9, 10, 11 ^]^ photovoltaic devices,^[^ 12, 13, 14 ^]^ and integrated circuit devices.^[^ 15, 16 ^]^

Despite their many advantages, LSPR‐enhanced structures have a drawback in that their functions are predetermined and unchangeable under the given plasmonic nanostructures, limiting their versatility. Thus, to address the limitations of fixed plasmonic systems, researchers have investigated the possibilities of active and switching plasmonics since the early 2000s, leading to the emergence of switchable plasmonic nanostructures.^[^ ^17^ ^]^

Switchable plasmonic nanostructures offer the ability to control optical properties in a dynamic and reversible manner for real‐time operations, making them versatile tools for various applications. For instance, they can enhance biosensor sensitivity and selectivity by tuning the LSPR to match the absorption spectra of the target analytes at extremely low concentrations.^[^ 18, 19, 20, 21 ^]^ In energy applications, these structures can control the absorption and emission of light, allowing efficient harvesting and conversion of solar energy.^[^ 22, 23, 24, 25 ^]^ In the field of plasmonic devices, switchable plasmonic structures have been used to develop dynamic color displays with a wide color range.^[^ 26, 27, 28, 29 ^]^ Using switchable plasmonic structures makes it possible to achieve a subwavelength optical resolution with an unlimited lifespan of pixels in next‐generation displays. Another emerging advantage of switchable plasmonic nanostructures is their ability to implement multiple functions in one device, thus reducing the need for additional optical components and enabling unique light modulation, previously difficult in 3D holographic displays.^[^ 30, 31 ^]^ Despite these developments in switchable plasmonic systems, the design and fabrication of nanostructures capable of reversible control remains a challenging task because reversible control requires an understanding of the dependence of various manufacturing parameters, such as material, size, shape, geometry, and orientation for engineering unique and efficient nanostructures that can modulate plasmonic oscillations customizable for specific applications.

However, developing plasmonic nanostructures with different types of stimuli, such as electrical, thermal, and optical, is difficult and often demands the convergence of various disciplines into a multifunctional implementation. One of the main topics of recent research on multifunctional implementation is reconfigurable metasurfaces intended to overcome poor signal‐to‐noise ratio (SNR) and performance degradation due to thermal absorption losses in metal‐based plasmonic systems.^[^ 32, 33, 34, 35, 36 ^]^ While switching plasmonics and reconfigurable metasurfaces can control optical properties in real time, reconfigurable metasurfaces—often fabricated to avoid metal‐based materials—focus on far‐field control.^[^ 33, 36, 37, 38, 39 ^]^ In addition, while reconfigurable metasurfaces use an entire harmony of meta‐atoms with a self‐resonant structure, switching plasmonic nanostructures can modulate optical properties with a relatively simple set of structures.^[^ 37, 38, 39, 40, 41 ^]^ In general, switching plasmonic structures provide distinct features, such as a compact design that can be integrated with existing systems, making them a practical solution for various applications.

Numerous reviews^[^ 42, 43, 44, 45, 46 ^]^ have pioneered active plasmonics and metamaterials; however, only a few have summarized the specific use of plasmonic nanostructure‐based switching or described optically switched modulation. Thus, this review aims to provide a clear and comprehensive overview of state‐of‐the‐art switching plasmonics and engineering switchable plasmonic nanostructures.

Here, we review switchable plasmonic nanostructures, a set of plasmonic devices, and systems whose functions are modulated by tuning external environment using optical, mechanical, electrical, or chemical stimuli. Only studies that achieved reversible modulation of switchable plasmonic nanostructures have been summarized. This review is organized as follows. In Section 2, we present the fundamental principles of switching plasmonics, followed by a discussion of various methods used to modulate switchable plasmonic nanostructures and an outline of their design and fabrication methods (Section 3). Among the numerous studies, related advances in biomedical, energy, and device engineering applications have been particularly emphasized. Switching‐based biosensing and imaging techniques, including surface‐enhanced Raman spectroscopy (SERS), have received particular attention as killer applications of plasmonics. We also discuss light and energy harvesting, which are of significant interest. Finally, we summarize the applications of dynamic plasmonic devices actively used in color displays (Section 4) and conclude the article from the perspective of current challenges and opportunities.

## 2 Principles behind Plasmonic Switching

This section presents a simple theoretical framework that may be useful for designing and analyzing switchable plasmonic nanostructures. The principles of plasmonic switching can be understood by considering the underlying physical processes that govern the optical and electronic properties of plasmonic nanostructures. We first describe two fundamental surface plasmon (SP) modes, localized SP (LSP), and SPP, together with the photoinduced thermal energy, called plasmonic heating (Section 2.1). Classic theories of SPR are based on metals; therefore, the analysis here primarily focuses on SP excitation in metals, even though SP modes can also be derived from nonmetallic materials such as highly doped semiconductors and graphene.^[^ 1, 2, 43, 47, 48 ^]^ Plasmonic nanostructures possess several geometric and structural parameters that can be engineered, opening up the possibility of exciting and controlling the LSP and SPP, which can be coupled to form hybrid optical modes, such as the gap mode and one associated with surface lattice resonance (SLR), for efficient optimization of performance (Section 2.2).^[^ ^48^ ^]^ The intrinsic nature of plasmonic nanostructures can also be modulated to attain tunability, with phase transitions and stimuli‐responsive materials adopted as working strategies (Section 2.3). This chapter comprehensively explains the theoretical correlation between the SP modes and the variables that could influence them, which may help readers understand the numerous methods and strategies employed in designing switchable plasmonic nanostructures, as described in Section 3.

### 2.1 Basic Mechanism of SP Excitation

#### 2.1.1 SPP Wave

**Figure 1:**
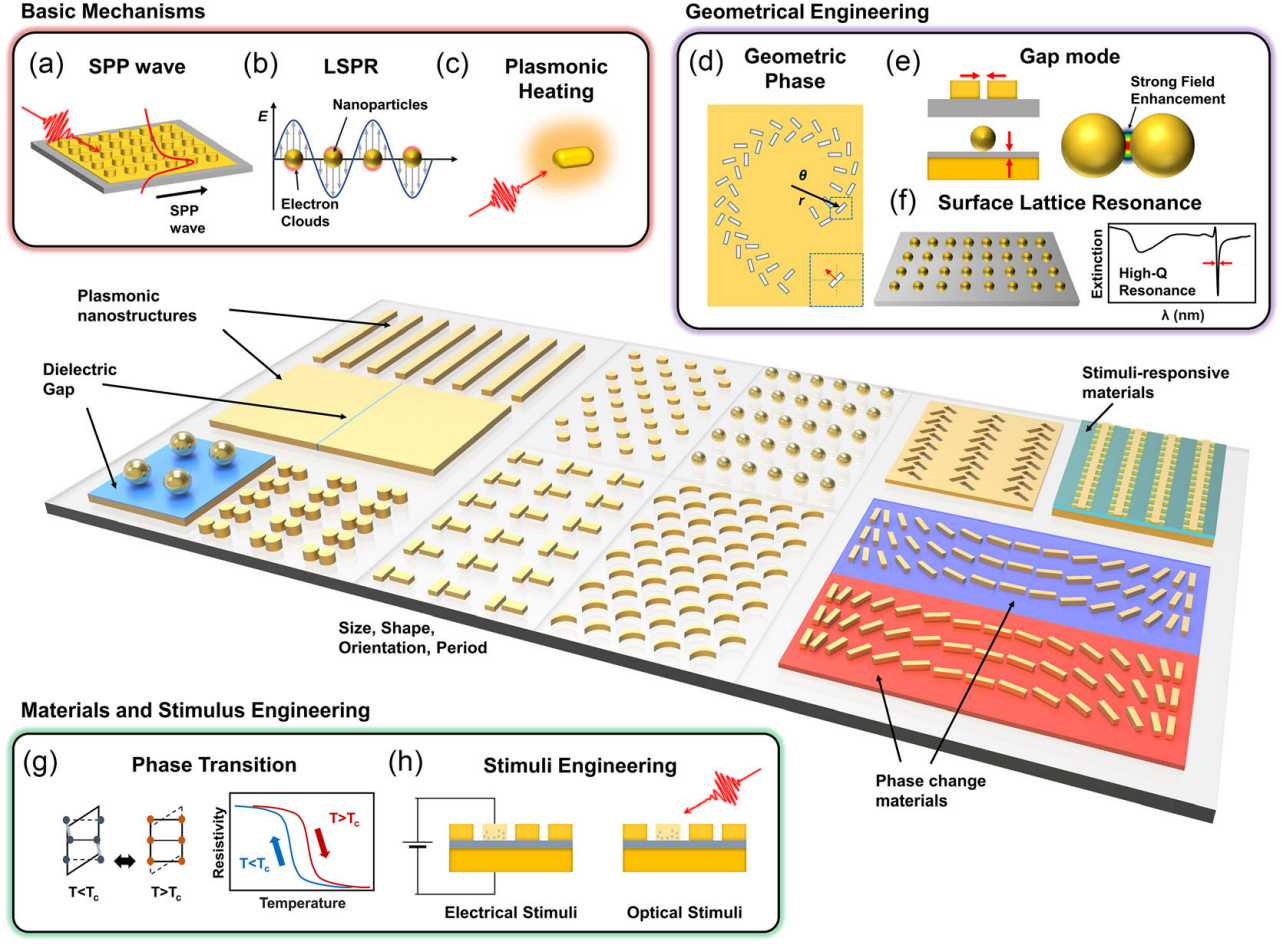
Principles behind plasmonic switching. a–c) Basic mechanisms: a) SPP wave propagates on surface of plasmonic array. b) LSPR occurs in metallic NPs of subwavelength size. c) Plasmonic heating occurs when plasmonic NPs absorb light and convert it into heat. Geometrical engineering. d) Geometric phase occurs when surface wave propagates in nonuniform or curved medium, including curved metallic surfaces or specifically arranged nanostructures. e) Gap mode occurs in narrow gap between two closely spaced metallic structures, such as a metal particle and metal film or two closely packed NPs. f) SLR occurs in ordered array of metallic NPs, leading to collective resonance with high sensitivity to lattice geometry. Materials and stimulus engineering. g) Phase transition can be induced or controlled by temperature or other stimuli. h) Stimuli engineering involves designing and manipulating plasmonic structures to respond to specific stimuli, such as light, temperature, or applied voltage.

SPP represents light‐coupled 2D charge‐density waves propagating along the metal–dielectric interface. Owing to the discontinuity in electric permittivity across the interface, its characteristics lead to strong axial confinement and enhancement of the electromagnetic field intensity.^[^ 1, 2 ^]^ The dispersion relation of the SPP on a flat metal–dielectric interface relates the wave vector ($k_{\text{SPP}}$) to the properties of the metallic and dielectric materials and is expressed as^[^ 1, 2 ^]^

(1) $$k_{\text{SPP}}=k_{0}\sqrt{\frac{\varepsilon_{d}\varepsilon_{m}}{\varepsilon_{d}+\varepsilon_{m}}}$$

where $k_{0}$ is the wave vector of the incident light in free space and $\varepsilon_{d}$ and $\varepsilon_{m}$ are the dielectric constants of the dielectric and metal film, respectively. The SPPs have a higher in‐plane momentum $k_{\text{SPP}}$ than $k_{0}$ in free space; this momentum mismatch between the incident light and SPP wave can be overcome with additional momentum.^[^ 1, 2, 43 ^]^ One method for exciting an SPP mode with light in free space is to use plasmonic nanoparticle (NP) array with a periodic arrangement.^[^ 49, 50 ^]^ For the simple 2D metal array shown in **Figure** **1**a, the following condition must be satisfied for SPP excitation.

(2) $$k_{\text{SPP}}=k_{0}\text{sin}\theta \pm iG_{x}\pm jG_{y}$$

where *θ* is the incident angle, *i* and *j* are integer pairs, and $G_{x}$ and $G_{y}$ denote the reciprocal vectors of the array, whose magnitudes are given by $G_{x}=G_{y}=2\pi /a$ (*a* is the period of the array). From Equation (1) and (2), it is clear that the SPP propagation can be modulated by the angle of incidence as well as by the period and morphology of the plasmonic NP arrays.^[^ ^48^ ^]^

#### 2.1.2 Localized Surface Plasmon Resonance

LSPs are nonpropagating excitations of conduction electrons in metallic nanoscale structures, such as NPs, coupled to an electromagnetic field. These modes arise naturally from the scattering of subwavelength conductive NP in an oscillating electromagnetic field.^[^ 1, 47, 51, 52, 53 ^]^ In a plasmonic NP array, individual NPs typically support LSP modes, where free electrons collectively oscillate with incident light at a specific frequency (Figure 1b). The LSPR frequency is determined by the size, shape, and composition of the NPs.^[^ 1, 48 ^]^ In addition to modifying the geometrical properties of NPs, the LSPR frequency can be tuned by modulating the dielectric ambiance.^[^ 7, 43, 48, 54, 55 ^]^ Changes in the refractive index of the surrounding medium or the addition of molecules that can bind to the NP surface and alter its properties can lead to alterations in the LSPR intensity, spectral shape, and polarization, all of which can be exploited for various applications.^[^ 51, 52, 56, 57, 58, 59 ^]^

One way to measure the strength of LSPR is through the extinction cross section ($C_{\text{ext}}$) of NPs, which represents the ability to absorb and scatter light; $C_{\text{ext}}$is the sum of the absorption and scattering cross sections ($C_{\text{abs}}$ and $C_{\text{sca}}$).^[^ 1, 48 ^]^ The cross section of a homogeneous and isotropic nanosphere (radius a≪λ) can be calculated using the following equations

(3) $$C_{\text{sca}}\left(\omega \right)=\frac{k^{4}}{6\pi }{\left|\alpha \right|}^{2}=\frac{8\pi }{3}k^{4}a^{6}{\left|\frac{\varepsilon_{m}\left(\omega \right)-\varepsilon_{d}\left(\omega \right)}{\varepsilon_{m}\left(\omega \right)+2\varepsilon_{d}\left(\omega \right)}\right|}^{2}$$

(4) $$C_{\text{abs}}\left(\omega \right)=k\text{Im}\left[\alpha \right]=4\pi k{\text{ a}}^{3}\text{Im}\left[\frac{\varepsilon_{m}\left(\omega \right)-\varepsilon_{d}\left(\omega \right)}{\varepsilon_{m}\left(\omega \right)+2\varepsilon_{d}\left(\omega \right)}\right]$$

(5) $$C_{\text{ext}}=C_{\text{abs}}+C_{\text{sca}}$$

where *k* and *ω* are the wavenumber and angular frequency of the incident light, respectively. Im(α) is the imaginary part of the polarizability of the NP. Even though Equation ((3), (4))–(5) were derived for homogeneous metal nanospheres, the general principle of LSPR remains valid for plasmonic nanostructures comprising NPs of different shapes with complex arrangements.^[^ 1, 48 ^]^

#### 2.1.3 Plasmonic Heating

Upon exposure to light energy, plasmonic nanostructures induce light–matter interactions that enhance the extremely localized electromagnetic field and produce heat (Figure 1c); this phenomenon is called plasmonic heating, which, in principle, originates from Joule heating.^[^ 60, 61, 62, 63, 64 ^]^ Plasmonic heating involves the conversion of absorbed light to thermal energy, which is dissipated partly into the surrounding medium. Despite its significance, plasmonic heating has historically been overlooked in many studies. However, recently, there has been a growing interest in the application of plasmonic heating in various fields (often referred to as thermoplasmonics).^[^ 60, 65 ^]^

In the case of an NP, the absorbed power due to plasmonic heating can be expressed using the absorption cross‐section $C_{\text{abs}}$ introduced in Section 2.1.2.^[^ 60, 64, 66 ^]^

(6)

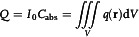

where $I_{0}$ is the intensity of the incident light and q(r) is the heat power density. Owing to Joule effects, the heat power density can be expressed using the following equation.

(7) $$q\left(r\right)=\frac{1}{2}\text{Re}\left[J{\left(r\right)}^{*}\times E\left(r\right)\right]=\frac{\omega }{2}\varepsilon_{0}\text{ Im}\left\{\varepsilon_{m}\left(\omega \right)\right\}{\left|E\left(r\right)\right|}^{2}$$

$J\left(r\right)=i\omega \alpha =i\omega \varepsilon_{0}\varepsilon_{m}\left(\omega \right)E\left(r\right)$ represents the complex amplitude of the current density inside the NP. The behavior of heat around plasmonic nanostructures can be described by the general heat transfer equation, as shown below.

(8) $$\rho C_{s}\frac{\partial T}{\partial t}-\nabla \times \left[\kappa \nabla T+h_{\text{cv}}\left(T_{0}-T\right)+\varepsilon_{\text{em}}\sigma \left(T_{0}^{4}-T^{4}\right)\right]=q$$

where *ρ* and $C_{s}$ are the density and specific heat capacity of the material, respectively, *κ* depicts thermal conductivity, and $h_{\text{cv}}$ represents the heat transfer coefficient for convection. $T_{0}$, $\varepsilon_{\text{em}}$, and *σ* are ambient temperature, surface emissivity of plasmonic material, and Stefan–Boltzmann constant, respectively. Plasmonic heating can also act as one of the principles underlying plasmonic switching, which may lead to a shift in the LSPR wavelength by inducing a change in the plasmon resonance of NP.^[^ 64, 67, 68, 69 ^]^

### 2.2 Geometrical Engineering

#### 2.2.1 Geometric Phase Control

In terms of controlling the SPs, the geometric phase refers to the phase shift that occurs when a wave travels in a curved or nonuniform environment, such as a curved metallic surface or a specially arranged group of nanostructures (Figure 1d).^[^ 70, 71, 72, 73 ^]^ Engineering the geometry of a plasmonic nanostructure makes it possible to control the geometric phase and hence the polarization and phase of the plasmonic mode through a selective plasmonic response, which can be used to switch between different plasmonic modes or turn the plasmonic response on and off. These modifications have been effectively demonstrated in various plasmonic nanostructures, including nanoslit arrays^[^ 71, 73, 74, 75, 76 ^]^ and curved structures.^[^ 77, 78 ^]^

#### 2.2.2 Gap Mode

Gap mode plasmonics refers to the interaction of plasmon between two closely spaced metallic NPs, resulting in strong field enhancement and confinement within a nanometer‐sized gap (Figure 1e).^[^ 79, 80, 81, 82, 83, 84 ^]^ Owing to its high signal enhancement and drastic changes in response to changes in the gap distance and surrounding dielectric environment, gap mode plasmonics is widely used in plasmonic sensing and imaging applications, for example, SERS analysis,^[^ 85, 86, 87, 88, 89, 90, 91, 92 ^]^ which may produce ultrahigh sensitivity even down to the detection of single molecules. In addition to sensing and imaging, the gap mode effect can be utilized for plasmonic switching by controlling the separation between NPs, accomplished via various external stimuli, for example, by introducing a bridging molecule or conjugated material that can regulate the gap distance, which results in the suppression or enhancement of the gap mode resonance, thereby achieving plasmonic switching.

#### 2.2.3 Surface Lattice Resonance

The SLR is a type of plasmonic resonance that occurs in a 2D periodic array of plasmonic NPs. In the SLR, the plasmons in each NP interact to produce a collective resonance localized at the surface of the array.^[^ 93, 94, 95 ^]^ Compared to LSPR, SLR possesses significant potential for high‐Q resonance (Figure 1f) compared to LSPR, which is of great interest in areas such as plasmonic lasing and sensing.^[^ 96, 97, 98, 99, 100, 101 ^]^ Coupled dipole approximation, in which an array of *N* NPs is replaced with an array of electric dipoles,^[^ 93, 95, 99, 102, 103 ^]^ allows the strength of the SLR to be characterized using the extinction cross section, $C_{\text{ext}}$, which describes the efficiency of the NP array to scatter and absorb light. The extinction cross section can be calculated using the same expression as that for individual NPs, but with a modified polarizability that considers the electromagnetic interactions between the NPs.

(9) $$C_{\text{ext}}=4\pi k\text{ Im}\left(\alpha_{\text{eff}}\right)$$

where $\alpha_{\text{eff}}$ is the effective polarizability of the NP array, dependent on the size, spacing, and dielectric properties of the NPs and surrounding medium. Thus, the SLR can be suppressed or enhanced to achieve plasmonic switching by controlling the periodicity of the NP array.

### 2.3 Materials and Stimulus Engineering

#### 2.3.1 Phase Transition

Phase transition is a physical process that causes changes in the intrinsic properties of surrounding materials, which can be exploited for plasmonic switching. Phase transition materials or phase‐change materials undergo a reversible change between the insulator and metal or between amorphous and crystalline states in response to external stimuli such as heating,^[^ 104, 105, 106, 107, 108 ^]^ optical absorption,^[^ 109, 110, 111, 112, 113 ^]^ or electric fields.^[^ 114, 115, 116 ^]^ This phase transition can lead to changes in the optical properties of a material, including its refractive index and absorption properties, resulting in a switchable plasmonic response. Vanadium dioxide (VO_2_) is an emerging photothermal material. At low temperatures, VO_2_ is in an insulating phase and does not interact strongly with light. In contrast, at higher temperatures, VO_2_ transitions to the metallic phase, significantly changing its optical properties (Figure 1g), including a shift in its plasmon resonance wavelength.^[^ 117, 118, 119, 120 ^]^ This change in the plasmon resonance wavelength can be used to achieve plasmonic switching. Similarly, other phase transition materials such as chalcogenides^[^ 121, 122 ^]^ and liquid crystals^[^ 123, 124, 125, 126 ^]^ can also be used to achieve plasmonic switching.

#### 2.3.2 Stimulus‐Responsive Materials

The use of stimuli‐responsive materials is another fundamental principle underlying plasmonic switching. They undergo a reversible change in free electrons and photocarrier excitation in response to external stimuli, such as optical absorption and electric fields. Dynamic modulation of plasmonic activity can be achieved by exploiting changes in the intrinsic properties (Figure 1h).^[^ 127, 128, 129, 130 ^]^ One such material is indium tin oxide (ITO), widely used in semiconductor devices such as flat‐panel displays and touch screens due to its unique properties.^[^ 131, 132, 133, 134 ^]^ ITO is a transparent conducting electrode with a high refractive index and can conduct electricity, attributed to the addition of tin to indium oxide, which introduces free electrons into the material and makes ITO conductive. Simultaneously, the oxygen ions in the material provide the ITO with a high refractive index, allowing it to function as a transparent material. By applying an electrical bias between the metal and ITO, the real part of the dielectric permittivity of the ITO in the accumulation layer ($\varepsilon_{\text{ITO}}$) can be changed from positive to negative. When $\left|\varepsilon_{\text{ITO}}\right|$ is in the epsilon‐near‐zero (ENZ) region, the accumulation layer experiences a significant electric field enhancement at near‐infrared wavelengths,^[^ 135, 136, 137, 138 ^]^ thus providing an effective method to electrically modulate the optical characteristics of nanophotonic devices with a high modulation speed and low power consumption. For example, ITO can be used as a thin‐film substrate for metal nanostructures, such as gold nanorods (NRs)^[^ ^139^ ^]^ or gold gratings.^[^ 138, 139, 140 ^]^ When a voltage is applied to ITO, its refractive index can change, shifting the plasmon resonance of the metal nanostructure.

## 3 Methods and Strategies of Switching

With the increased understanding of plasmonics over the past few decades, the ability to control and manipulate plasmonic signals using external stimuli has led to the development of various methods and strategies for achieving switchable plasmonic nanostructures. In the following sections, we discuss the three main approaches for plasmonic switching: passive optical switching, an approach tunable with active structures, and one based on active materials. Through reviews of many works taking each of these approaches, we aim to provide insights into the current state of the art in plasmonic switching and highlight potential directions for future research.

### 3.1 Passive Optical Switching

One of the most straightforward and efficient methods for achieving plasmonic switching is passive optical switching, which operates without external excitation and instead relies on the plasmonic nanostructure and the intrinsic properties of an incident beam. In contrast to active plasmonic switching, passive optical switching depends on the intrinsic properties of the nanostructure, specifically the size, shape, and composition, to tune its plasmonic response. By changing these structural parameters, the resonant frequency of the nanostructure can be shifted, leading to changes in its optical properties. Furthermore, the optical properties of passive plasmonic structures can be modulated by changing the incident light parameters such as incident direction, polarization, and wavelength. This modulation of optical parameters allows the function of the nanostructure to be turned on or off or to be optimized for the desired applications. Although fabricated nanostructures may remain fixed, the optical properties of the incident light interacting with the structure can be altered to achieve various functions for control such as SPP wave, LSPR‐induced near‐field distribution, localized heating, and several far‐field properties including chirality.

Passive optical switching differs from the binary approach of general plasmonic systems, which excites SP when specific optical conditions are satisfied and turns off otherwise. In contrast, passive optical switching systems still rely on the intrinsic properties of the nanostructure and the on‐states of SP are multiconditioned and can have multiple possibilities, depending on the characteristics of the incident light. Passive optical switching systems represent the branches that have pushed the boundaries of plasmonics, highlighting a clear departure from typical plasmonic systems.

#### 3.1.1 SPP Switchable Nanostructure

**Figure 2:**
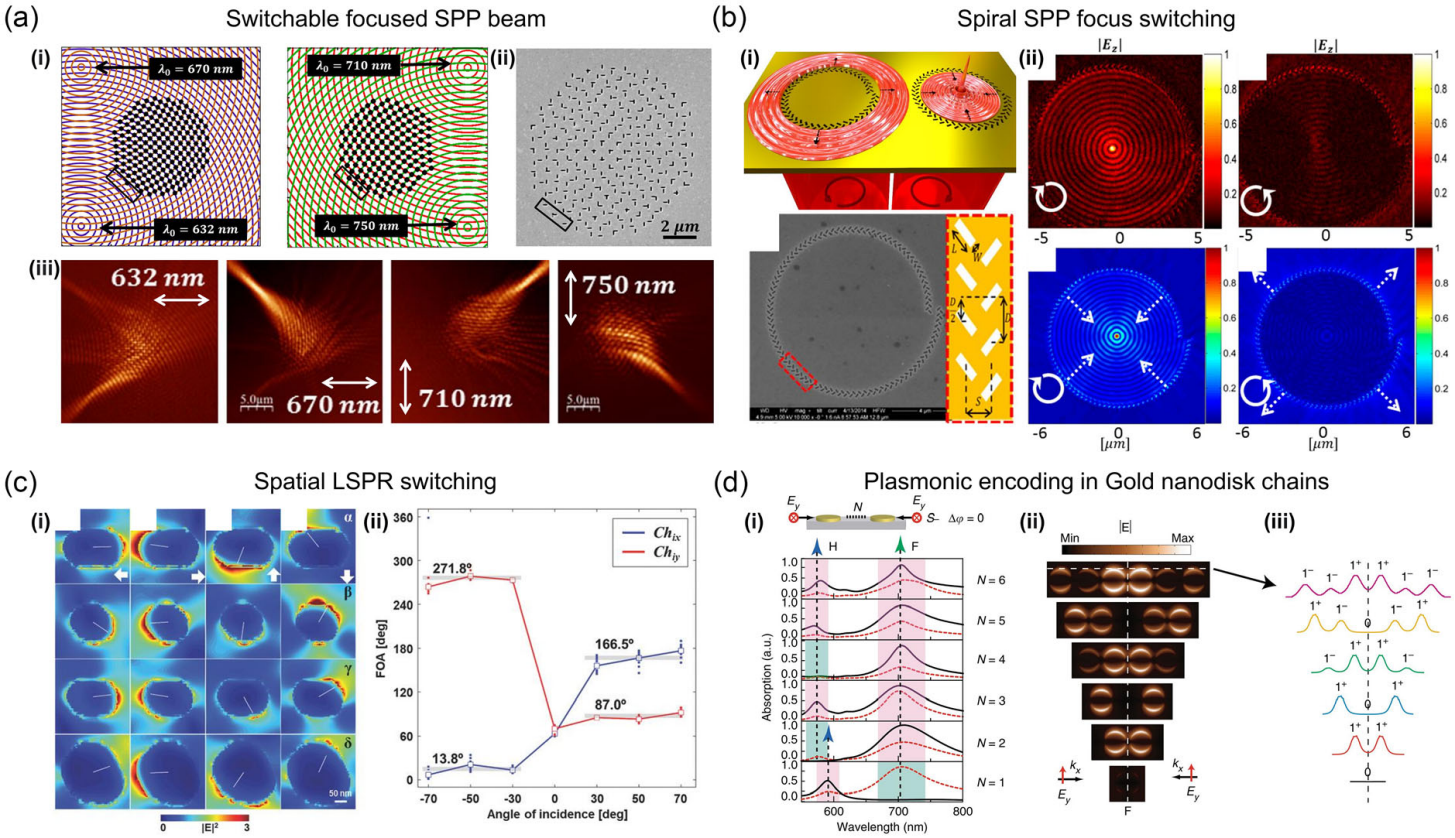
Tunable SPP focus and LSPR. a) Switchable metalens design. i) The metalens can focus light of four different wavelengths onto different corners of a 16 μm × 16 μm square. All of the scattered SPs that reach the respective focal point will be in phase because by design they lie on the equiphase lines. The wavefronts of an imaginary circular point source are represented by colored circles (632 nm, 670 nm, 710 nm, and 750 nm). The positions where vertical and horizontal nanoslits are milled are marked by black dots. A scanning electron microscopy (SEM) image of the metalens is shown in (ii). iii) Experimental near‐field scanning optical microscopy (NSOM) results with the polarization of light denoted by the white arrow. Depending on the wavelength and polarization, SPP beams are unidirectionally focused on the four corners of a square. a) Reproduced with permission.^[^ ^74^ ^]^ Copyright 2015, American Chemical Society. b) Spiral plasmonic metalens. i) A structural schematic and SEM image of a spiral plasmonic lens. ii) Experimental NSOM results and corresponding finite‐difference time‐domain method (FDTD) simulations of the spiral plasmonic lens under circularly polarized illumination denoted by the white arrows. SPP propagation direction inward and outward is shown by the dotted arrows. b) Reproduced with permission.^[^ ^75^ ^]^ Copyright 2015, American Chemical Society. c) Spatial LSPR switching on gold nanoislands. Calculated near‐field distribution in (i) as the four‐channel incident light is rotated at an angle of incidence (*θ*  = 70° and −70° for *x* and *y* incidence directions). The near‐field intensity distribution's direction is indicated by the white lines. The four different nanoislands are shown as rows of (*α*–*δ*). ii) Field orientation angle of a LSPR produced in the 14‐channel modes. c) Reproduced with permission.^[^ ^153^ ^]^ Copyright 2018, Wiley‐VCH. d) In‐plane coherent control of plasmon resonances. i) Calculated absorption spectra of gold nanodisk chains with varying numbers of nanodisks illuminated by the asymmetrical *s*‐polarized in‐plane plan illumination (dashed line) or symmetrical *s*‐polarized in‐plane plan illumination (solid line). The nanodisk has a 140 nm diameter, and the separation is 30 nm. The colors green and red, respectively, stand in for the destructive and constructive plasmon resonances. ii) Spatial distributions of E‐field amplitude under symmetrical illumination. iii) E‐field amplitude distributions along the edge of gold nanodisk chains (the white dashed line in (ii)). d) Reproduced under the terms of the CC‐BY Creative Commons Attribution 4.0 International license (https://creativecommons.org/licenses/by/4.0).^[^ ^158^ ^]^ Copyright 2019, The Authors, published by Springer Nature.

The process of coupling light with SPP in the thin‐film interface and controlling it has traditionally been challenging due to the sensitivity to polarization of the coupling efficiency and the challenge of managing the directional aspect of SPP.^[^ 141, 142, 143, 144, 145, 146 ^]^ It has been demonstrated that much flexible SPP control within a flat surface can be achieved using special components intrinsic to the surface, as follows, for example, Wintz et al.^[^ ^74^ ^]^ developed a novel metalens that provides switchable control of SPP focusing. They presented a metalens design strategy that overcomes various coupling and focusing issues associated with SPP. This design strategy provides both wavelength and polarization tunability for SPP beam propagation and can focus SPP beams after coupling by recreating the wavefront of a point source. As depicted in **Figure** **2**a, coupling free‐space light to nanostructured surfaces via subwavelength slits steers light along different directions depending on its wavelength and polarization, resulting in a focused SPP beam.

In a similar way, Spektor et al.^[^ ^75^ ^]^ developed a spiral plasmonic lens that can address the problems of efficiency and functionality encountered by standard in‐plane plasmonic lenses by combining global and local geometries. As shown in Figure 2b, the spiral plasmonic lens distinguishes between right‐ and left‐circularly polarized illumination, providing optimized focusing for matched circular polarization. Moreover, it directs all of the power coupled to SPP into the focal spot under matched circularly polarized illumination, achieving two orders of magnitude difference in intensity across the whole expanse of the lens. This research suggests that the integration of spirality and metasurface should result in significantly improved contrast and large‐area functional focusing of SPP while taking into account the geometric phase.

#### 3.1.2 Localized Near‐Field Switchable Nanostructure

In addition to the directional coupling and focusing of SPP, plasmonic nanostructures can also exhibit LSPR, which can be used to manipulate near‐field properties such as the electric field intensity and distribution. Numerous studies based on the use of metallic nanostructures for extreme light localization, including NR array^[^ 147, 148 ^]^ and nanogratings,^[^ 58, 59, 149, 150, 151, 152 ^]^ have explored ways to control the properties of LSPR. The flexible switchability can be achieved by controlling the parameters of nanostructures and incident light described in Section 2.

One notable example is the nanostructure for spatial light switching of LSPR, which allows for dynamic tuning of near‐field distribution. Son et al.^[^ ^153^ ^]^ investigated the use of random composite nanoislands for spatial light switching, which have become a subject of great interest for various applications due to the simple fabrication process such as temperature annealing. The near‐field characteristics of the random nanoislands produced by spatial light switching were examined using 14 incident channel modes to switch light incidence and polarization (−70°, −50°, −30°, 0°, 30°, 50°, 70° for *x* and *y* incidence directions). Experimental validation and numerical calculation indicate that the optical field localized at each nanoisland component is almost linearly displaced from the nanoisland with the polar incidence angle of the channels, as shown in Figure 2c. Additionally, the localized near‐field rotates significantly with the azimuthal direction of light incidence.

Not only switching in a single nanostructure but high‐order switching via coupling with surrounding nanostructures is also actively studied. One of the recent topics of research has focused on coherent management of plasmon resonance in metadevices for purposes such as plasmonic imaging,^[^ ^154^ ^]^ nanolasing,^[^ ^155^ ^]^ and optical communication.^[^ 156, 157 ^]^ Jiang et al.^[^ ^158^ ^]^ showed that in‐plane coherent control can be achieved by exploiting the distribution rules of electric field components of nanoantennas, which enabled the implementation of destructive and constructive absorption in metal nanoantennas and demonstrated the coherent management of plasmon resonance when exposed to symmetrical in‐plane lighting. As shown in Figure 2d, they proposed plasmonic encoding with a chain of gold nanodisks. Because of phase‐dependent coherent absorption throughout the chain, the constructive fundamental plasmon resonance of a gold nanodisk chain exhibits a clear spatially selective characteristic. In‐plane coherent management of plasmon resonance is highly dependent on the arrangement and symmetry of plasmonic nanostructures, allowing greater flexibility in customizing and manipulating various plasmon resonances within axisymmetrically shaped plasmonic nanostructures.

#### 3.1.3 Far‐Field Switchable Nanostructure

**Figure 3:**
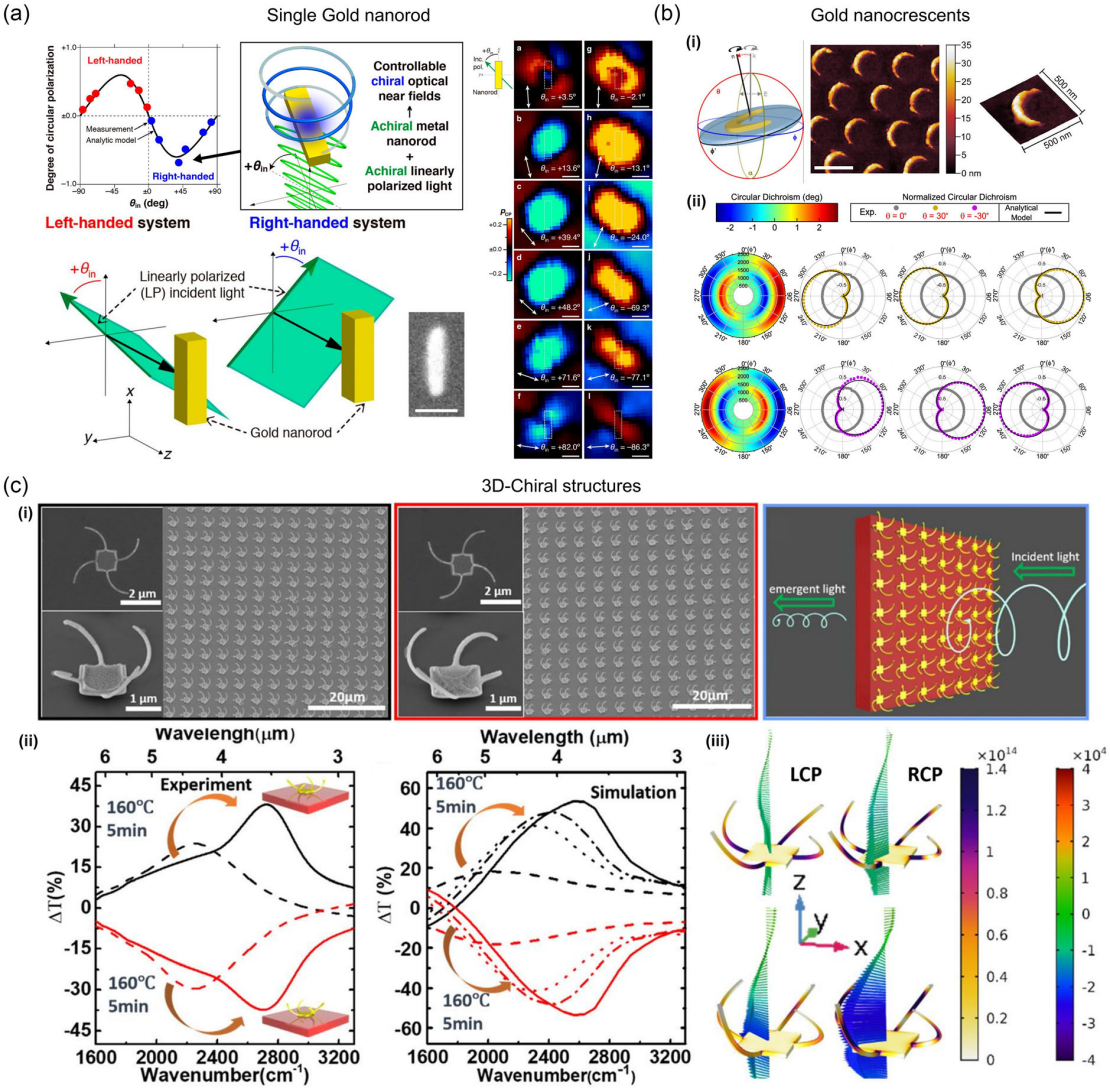
Far‐field chirality switching. a) Schematic images of the active control of the local chiral optical field around a gold NR by adjusting the azimuth angle of the incident linearly polarized optical field relative to the axis of the NR. The degree of asymmetry for the whole system, consisting of the NR and incident field, could be tuned, as shown in the schematic images. The inset SEM image shows a gold NR (160 nm × 40 nm × 55 nm) used in this study. Experimental maps for the degree of circular polarization of the optical fields near a single gold NR were observed by exciting it with a linearly polarized optical field with positive and negative azimuth angles. Scale bars of 100 nm are shown in both the schematic and experimental images. a) Reproduced with permission.^[^ ^162^ ^]^ Copyright 2019, American Chemical Society. b) Gold nanocrescents structural and chiral features. i) Schematic illustration of rotations applied in the orientation‐dependent extinction experiments. Gold nanocrescents topography taken by atomic force microscopy (AFM) with 500 nm scale bars. ii) Circular dichroism responses from circularly polarized light where the sample is rotated. For the polar contour plots at *θ* = 30°, −30°, the radial axis is the wavelength range 500–2500 nm. Polar plots show respective peak wavelength circular dichroism profiles at *θ* = ± 30° for the short‐axis backbone dipole mode (590 nm), the short‐axis tips dipole mode (1300 nm), and the long‐axis dipole mode (2200 nm). Polar plots for peak resonances are normalized within each respective wavelength set. b) Reproduced with permission.^[^ ^163^ ^]^ Copyright 2020, American Chemical Society. c) Thermomechanically configurable 3D chiral metamaterials. i) SEM images of the 3D chiral metamaterials. The left upper panel shows the top view of the planar meta‐atoms before release; the lower panel shows 52° tilt views after release to form the 3D meta‐atoms. The right panel shows a schematic of the metamaterials illuminated by circularly polarized light. ii) The left plot depicts experimental spectra of the transmission difference between right‐circularly polarized and left‐circularly polarized light. The right plot shows electromagnetic simulations of the spectra for the same structures. The different curves represent the sample before annealing (dashed), with experimental dielectric functions after annealing (dotted). iii) Calculations of the evolution of absorption and scattering to the incident light before (upper panel) and after (lower panel) annealing. The left and right color bar represents energy dissipation density and relative electric field, respectively. c) Reproduced with permission.^[^ ^164^ ^]^ Copyright 2020, American Chemical Society.

Chiral optical fields, characterized by circularly polarized light, have been shown to enhance chiral light–matter interactions at nanoscale, opening up potential novel applications of far‐field switching.^[^ 159, 160, 161, 162, 163, 164 ^]^ To realize these applications, it is essential to have a local chiral light source that can switch its chirality. Hashiyada et al.^[^ ^162^ ^]^ experimentally demonstrated the production and dynamic control of extensively chiral local optical fields via the use of achiral linearly polarized optical fields and achiral gold NRs. The adjustment of the azimuthal angle of incoming light with respect to the axis of the NR resulted in the generation of local optical fields with left‐ or right‐handed circular polarization, as shown in **Figure** **3**a. They demonstrated that chiral optical fields with high degree of circular polarization can be localized at the center above the NR by adjusting the relative angle between the long axis of the NR and the incident linear polarization. Since a single gold NR generates a continuously controllable chiral optical field, this technique allows a nanoscale polarization modulator to be implemented, enabling high‐sensitivity detection and characterization of chiral molecules.

Stevenson et al.^[^ ^163^ ^]^ introduced a method to manipulate active chiral plasmonic responses with substrate orientation control. Using gold nanocrescents, they have shown that changing the substrate orientation can control the degree of switching chirality and optical extinction. These plasmonic responses arise from the multipolar characteristic of resonant modes. By changing the incident light polarization, near‐field resonance can be selectively controlled, and substrate orientation can further manipulate this effect to produce dramatic changes in far‐field optical properties attributed to near‐field magnetoelectric dipolar and higher‐order multipolar coupling (Figure 3b). These findings have implications for the interpretation of optical scattering events in plasmonic systems as well as the nanostructure design and suggest potential utility for plasmon‐based magnetic field sensors and plasmonic magnetoelectric scatterers.

On the other hand, due to short optical path lengths of 2D chiral metasurfaces, the strength of the interactions between light and matter is frequently limited. As a 2D layer, they have inherent limitations that they cannot access 3D features such as intrinsic chirality. Previous nanofabrication has restricted the majority of optical metamaterial structures to a planar form, because simple 3D meta‐atoms require multiple patterning steps and have limited tunability. However, Guo et al.^[^ ^164^ ^]^ recently made a breakthrough using a scalable patterning technique in a single step that utilizes structural adaptability and chemical addressability of assemblies of colloidal gold nanocrystal. As shown in Figure 3c, optical properties were altered by inducing shape deformation in 2D nanocrystal/titanium bilayers with heat and directing their shape into complex 3D meta‐atoms. Specifically, through customization of the number, curvature, and length of 3D helix‐shaped structures in every meta‐atom, extensive metamaterials were produced showing chiral responses that exhibit a transmission variation between left‐hand and right‐hand circularly polarized light up to 40%. These 3D optical metamaterials are suitable for broadband circular polarizers and can switch the polarity of polarization rotation when the shape is thermally configured.

Overall, passive optical switching offers a simple yet effective method to modulate optical properties of plasmonic nanostructures. One of the significant advantages of passive optical switching is its low power consumption, making it a potential candidate for developing next‐generation optoelectronic devices. Moreover, the inherent properties of plasmonic nanostructures provide greater design flexibility and simplify the fabrication process, leading to cost‐effective and scalable production. With continued advancement in design techniques and modulation strategies, passive plasmonic structures hold great prospect for future applications in sensing, imaging, and energy harvesting. However, despite its numerous advantages, passive plasmonic switching still faces several challenges such as limited tunability, low switching speed, and material compatibility issues. Therefore, researchers continue to explore new materials and designs that can overcome these challenges and advance the field of plasmonic switching.

### 3.2 Active Structure‐Tunable Switching

The first step for active switching is to manipulate the spatial relationship between metals by applying an external force and maintaining internal properties of the metal nanostructure. Applied external forces include a macroscopic control that mechanically applies stretch and bending to the metallic array and an indirect control that applies external stimuli to the conjugated material and chemical structure with the metallic array. External stimuli can be used to tune the material. An external stimulus is not directly applied to the metallic nanostructure, but is intended to change the volume and arrangement of the surrounding material, which is clearly different from the material‐based switching described in Section 3.3. This section describes examples of tuning plasmonic response by changing the structural properties of nanostructures.

#### 3.2.1 Stretch‐ and Curvature‐Tunable Switching

**Figure 4:**
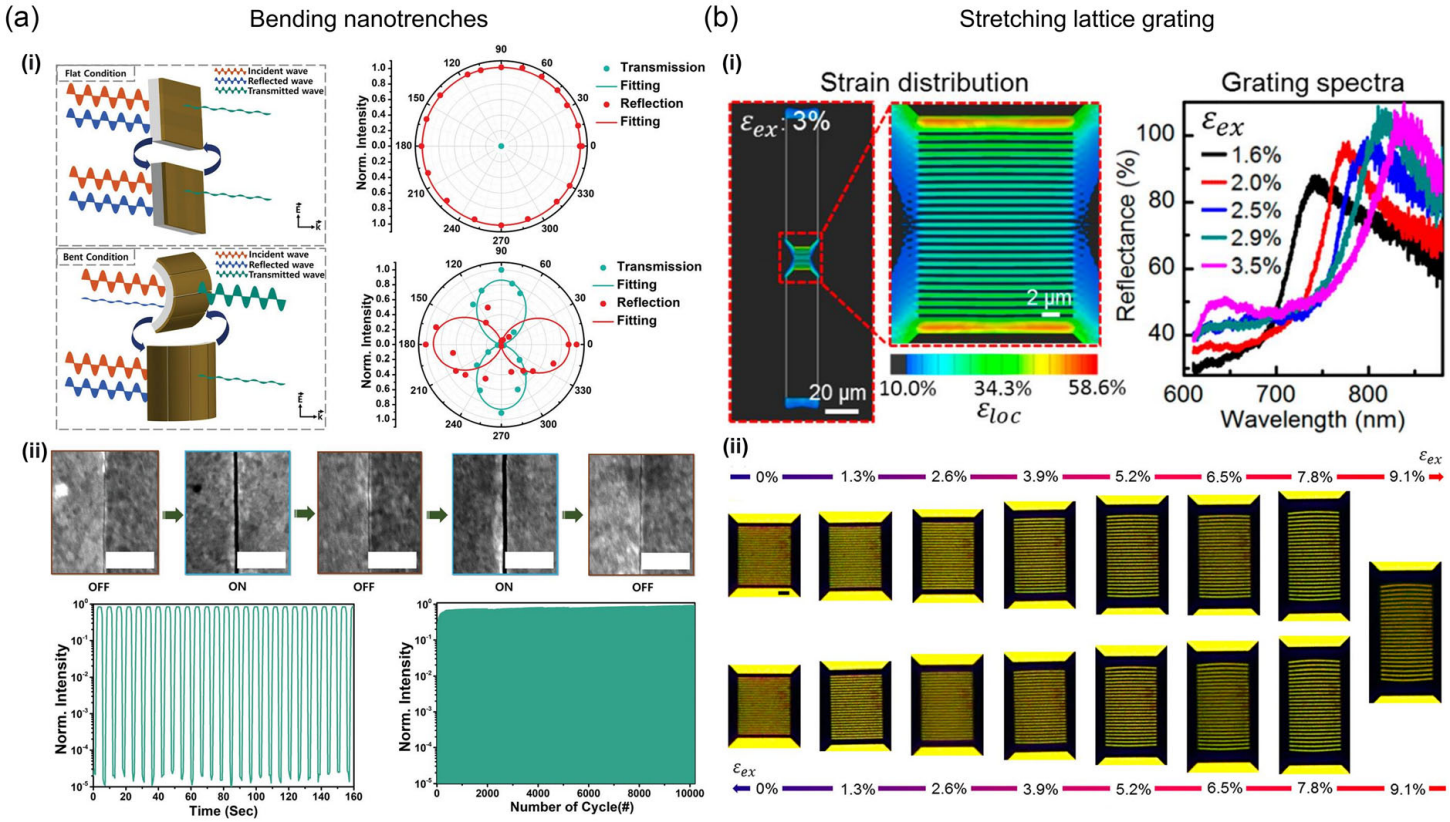
Stretch and curvature‐tunable switching. a) Bending zero‐gap‐embedded template. i) The schematic diagram on the left illustrates the concepts of a mirror for a flat zero‐gap‐embedded template and a polarizer for a bent zero‐gap‐embedded template. The right plot shows the polarizing ability of the zero‐gap‐embedded template. When the zero‐gap template is flat, it exhibits mirror‐like behavior, and when the metasurface is bent, it exhibits polarizer‐like behavior. ii) SEM images demonstrating a clear recovery of the gap after numerous cycles of bending and relaxing (scale bar: 1 μm), as well as microwave intensity modulation at 15 GHz with the repeated transformation between mirror and polarizer by bending and relaxing. An improved fatigue test with more than 10 000 repetitions is shown in the lower right. a) Reproduced with permission.^[^ ^165^ ^]^ Copyright 2021, Wiley‐VCH. b) Strain amplification on metastructure. i) Finite‐element modeling of the strain distribution on the metastructure on the surface of the PDMS substrate. The magnified view shows the strain distribution in the region of plasmonic grating. The color scale represents the local strain in the *y*‐direction on the PDMS surface. The right plot shows experimental reflectance spectra at normal incidence. ii) Optical microscopy images of the plasmonic grating under mechanical responses of continuous loading and unloading (scale bar: 2 μm). b) Reproduced with permission.^[^ ^169^ ^]^ Copyright 2018, American Chemical Society.

Mechanically bending the substrate changes the substrate properties physically and shifts plasmonic resonance, making it intuitive to apply to a variety of applications with nanotrenches,^[^ 165, 166 ^]^ split‐ring resonators,^[^ 166, 167 ^]^ and Bragg reflectors.^[^ ^168^ ^]^ Recently, Das et al.^[^ ^165^ ^]^ demonstrated a new type of multifunctional switchable metasurface with the ability to fully transform from a perfect mirror to a near‐transparent polarizer. The metasurface operates in the microwave regime and is activated by the zero‐gap technique, allowing prepatterned cracks in metal films to be activated and healed repeatedly, as shown in **Figure** **4**a. By transforming a bare metallic film into well‐controlled nanometer‐sized gaps in a fully reversible manner, researchers have achieved operation in a broad frequency range with a high extinction ratio and fatigue resistivity.

Followed by bending, stretching the substrate is easy to mechanically change the spatial relationship of a metallic array including lattice grating,^[^ ^169^ ^]^ nanodisks,^[^ ^170^ ^]^ self‐assembled nanodomes,^[^ ^171^ ^]^ and gold NR crosslinked polymer.^[^ 172, 173 ^]^ By controlling the distance of individual components, for example, by stretching plasmonic array, plasmonic response can be shifted by the change of SLR discussed in Section 2.2.3.

Chen et al.^[^ ^169^ ^]^ focused on the development of structurally reconfigurable optical metasurfaces capable of manipulating the resonant wavelengths of plasmonic gratings in a highly sensitive, linearly dependent, and reverse stretchable way. The use of microstructured poly(dimethylsiloxane) (PDMS) substrates and a plasmonic lattice array integrated between a pair of symmetric microrods, depicted in Figure 4b, allows the amplification of strain induced by external mechanical stimuli. Similar approaches have been taken to the development of ultrasensitive stretchable optics and electronics, including tunable metasurface,^[^ 174, 175 ^]^ plasmonic color pixels,^[^ 176, 177 ^]^ and nanolasers,^[^ 178, 179 ^]^ as well as wearable strain sensors and stretchable devices.^[^ 180, 181 ^]^

**Figure 5:**
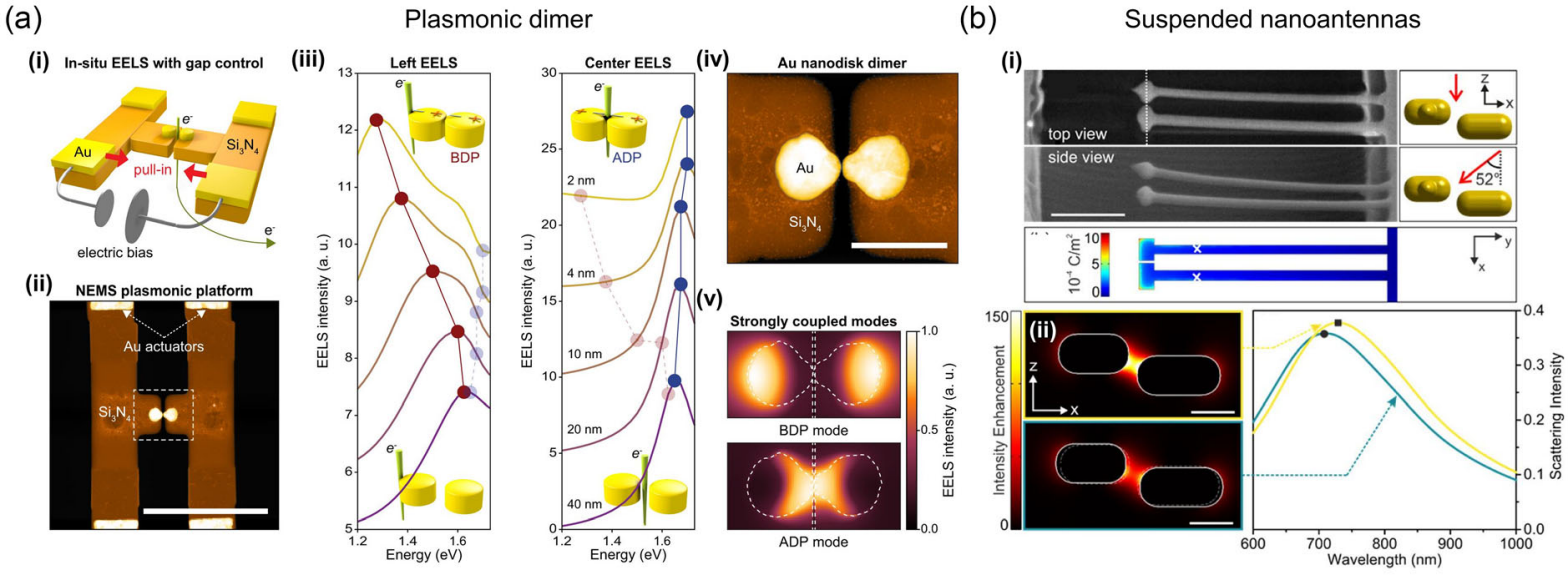
Electrostatic‐force‐induced mechanical switching. a) A NEMS that utilizes a plasmonic dimer. i) Schematic illustration of the device that operates by applying an external DC bias to actuators, which in turn pulls two Si_3_N_4_ beams together and reduces the spacing between two gold NPs. ii) The fabricated dimer is depicted in a false‐colored scanning transmission electron microscopy (STEM) image, with the dimer shown in white/yellow and the cantilevers in brown. A white, dashed rectangle indicates the location of the dimer (scale bar: 1 μm). iii) Simulated EELS spectra to investigate the impact of gap size on the electron beam incident on the left edge and center of a gold nanodisk dimer. They implemented a stepwise increase of offset values to enhance visibility. The red and blue symbols were used to denote the bonding dipole (BDP) and the antibonding dipole (ADP) modes, respectively. iv) They employed a magnified false‐colored STEM image to present a gold nanodisk dimer. The gold nanodisk was fabricated with a diameter of 100 nm and a thickness of 50 nm (scale bar: 200 nm). v) Simulated EELS spatial profile to examine the BDP and ADP modes for a 2 nm gap size. They incorporated a white dashed contour obtained from STEM image in (iv) into the EELS simulation. a) Reproduced under the terms of the CC‐BY Creative Commons Attribution 4.0 International license (https://creativecommons.org/licenses/by/4.0).^[^ ^184^ ^]^ Copyright 2021, The Authors, published by Springer Nature. b) Suspended optical nanoantenna for electromechanical tuning of plasmon resonance. i) SEM images (upper) taken in top and side views of the fabricated suspended optical nanoantenna. COMSOL simulated charge density distribution (lower) for a 10 V applied voltage on the antenna and lead wires. ii) Near‐field intensity distribution (left) and scattering spectra (right) of optical nanoantennas acquired by FDTD simulation. b) Reproduced with permission.^[^ ^185^ ^]^ Copyright 2016, American Chemical Society.

Electrostatic forces have been also exploited for a strategy of structure‐tunable plasmonic switching.^[^ ^182^ ^]^ Specifically, electrostatic forces have been used for actuation to manipulate plasmonic structures mechanically.^[^ ^183^ ^]^ Song et al.^[^ ^184^ ^]^ reported a nanoelectromechanical system (NEMS) to modulate the gap between plasmonic dimers. They achieved ≈10 MHz frequencies to operate plasmonic modulation with very low power consumption. They presented a modulated nanogap of dimers down to 1 nm and results were demonstrated using electron energy loss spectroscopy (EELS) (see **Figure** **5**a).

Similarly, Chen et al.^[^ ^185^ ^]^ highlighted the potential to realize large‐bandwidth optical NEMS by bending the flexible nanostructure itself. As shown in Figure 5b, a suspended two‐wire plasmonic nanoantenna acting like a nanoelectrometer was demonstrated. The antenna wires are supported and electrically connected via thin leads without disturbing the antenna resonance. By applying a voltage, equal charges are induced on both antenna wires, and the resulting equilibrium between the repulsive Coulomb force and the restoring elastic bending force allows precise control of the gap size. Hence, the resonant wavelength and electric field enhancement of the suspended optical nanoantenna are reversibly tuned.

#### 3.2.2 Chemical‐Assisted Structure‐Tunable Switching

Mechanical adjustment of the substrate or nanostructure has a relatively low degree of freedom and is difficult to achieve precise control. Several methods have been proposed to control with a high degree of freedom by connecting metallic NP–NP links with special materials and compounds while applying external stimuli.

In this context, DNA origami presents a popular technique due to its unique ability to precisely arrange and control the position and orientation of plasmonic NPs.^[^ 186, 187, 188, 189 ^]^ The high specificity and programmability of DNA origami make it an ideal platform for the design and assembly of complex plasmonic nanostructures with tailored optical properties. Kuzyk et al.^[^ ^190^ ^]^ demonstrated reconfigurable 3D plasmonic chiral metamolecules capable of executing switched chiral reactions via DNA‐assisted structural modulation. A switchable DNA origami template made up of two linked bundles was used to connect two gold NRs, allowing the angle between them to be adjusted. The 3D chiral nanostructure can be actively controlled using two DNA locks, which extend from the sides of the template. To alter the relative angle between the two DNA bundles and thus the gold NRs, specifically designed DNA strands were used as fuel to drive the plasmonic nanostructure into desired states. Recently, the same research group demonstrated a transformable plasmonic helix system, in which multiple gold NPs can be directly rearranged by DNA swing arms.^[^ ^191^ ^]^ These efforts offer a way to construct plasmonic systems with unique optical properties, using a method that involves cooperative rearrangement of optical components with a high degree of accuracy.

**Figure 6:**
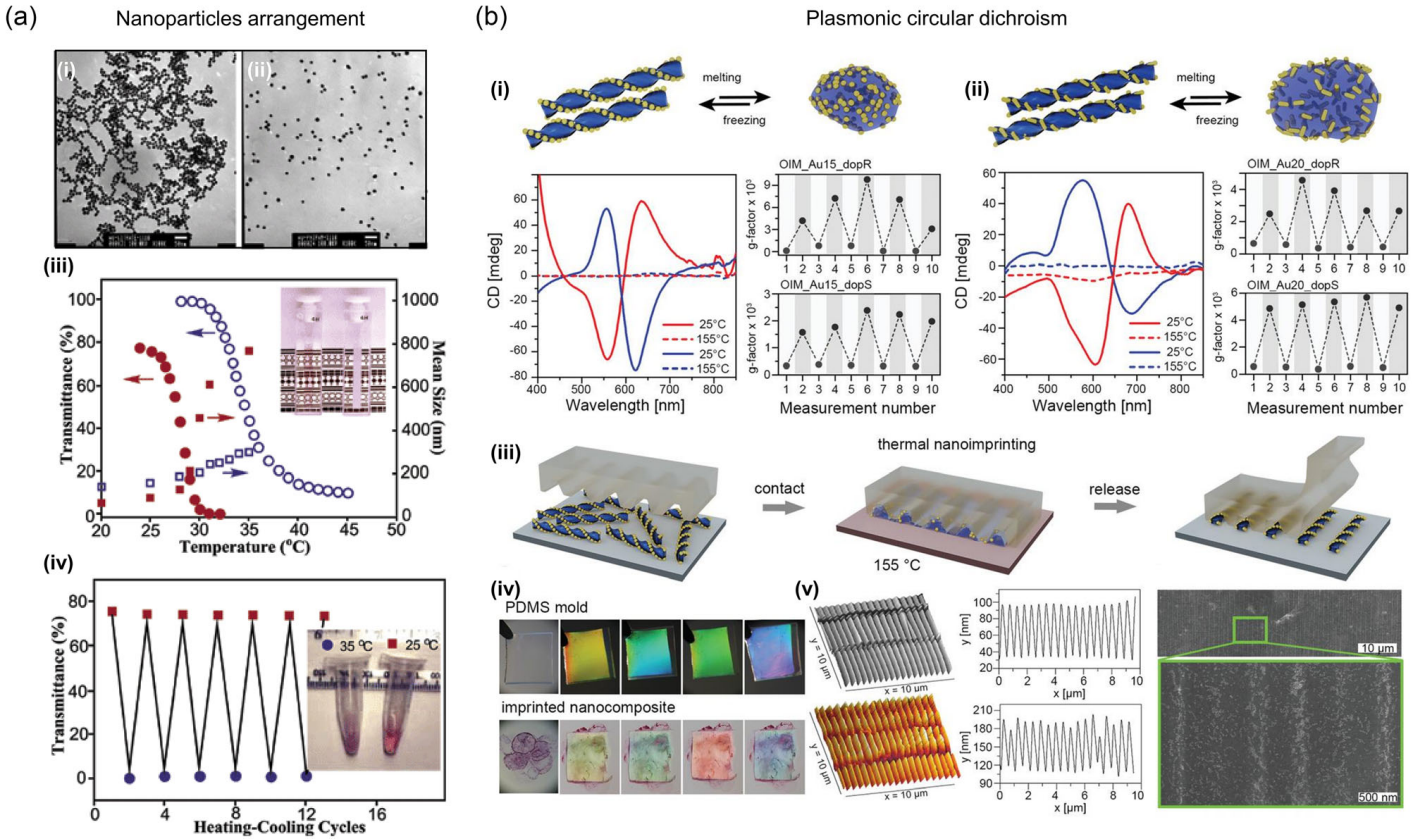
Chemical‐assisted plasmonic switching. a) Synthesis of thermosensitive polymers with gold NPs. i,ii) Transmission electron microscopy (TEM) images were taken of gold NPs that were citrate stabilized (i) and those that were PPA tethered (ii), which had a size of ≈13 nm. iii) The transmittance of PPA‐functionalized gold NPs (solid circles) and PPA (open circles) showed thermoresponsive changes that shifted from opaque to transparent. The mean diameter of the thermosensitive gold NPs (solid squares) was measured using dynamic light scattering and showed a more significant increase at the transition temperature point compared to the PPA polymer (open squares). iv) The solution was observed to switch reversibly from a slightly purple opaque suspension (with an gold plasmon resonance band at 566 nm that was very broad) to a transparent red solution (with a normal gold plasmon resonance band at 527 nm) between 35 °C (circles) and 25 °C (squares). a) Reproduced with permission.^[^ ^196^ ^]^ Copyright 2004, Wiley‐VCH. b) Fabrication of PCD thin films. i) Schematic model of liquid crystalline matrix based gold NP assemblies on thermal melting/freezing. Lower left: Circular dichroism spectra for each chiral‐selective state at 25 and 155 °C (solid and dashed lines, respectively). Lower right: maximum *g*‐factor value in consecutive heating/cooling cycles of each chiral‐selective state. ii) Diagram of thermally melted/frozen assemblies of liquid crystalline matrix‐based gold NRs. Lower left: Circular dichroism spectra at 25 and 155 °C for each chiral‐selective state (solid and dashed lines, respectively). Lower right: maximum *g*‐factor value during each chiral‐selective state's subsequent heating and cooling cycles. iii) Schematic model of the thermal nanoimprinting process. iv) Images of a wrinkled PDMS mold and a film made of liquid crystalline matrix based gold NP assemblies before and after the imprinting process, iridescence from light diffraction caused by the micropattern, viewed at various tilt angles. v) AFM topography images (left) of a patterned PDMS mold and liquid crystalline matrix based film after nanoimprinting, as well as corresponding surface height profiles. SEM images (right) of an imprinted sample. b) Reproduced with permission.^[^ ^195^ ^]^ Copyright 2022, Wiley‐VCH.

The combination of thermoresponsive materials and plasmonic NPs presents another fundamental approach for achieving structure‐tunable plasmonic nanostructures.^[^ 192, 193, 194, 195 ^]^ A commonly employed methodology involves the utilization of thermoresponsive molecules or polymers to modify NPs. One such example is poly(*N*‐isopropylacrylamide) (PPA), which has been extensively studied and exhibits phase transition from hydrophilic‐to‐hydrophobic states at a low critical solution temperature. Zhu et al.^[^ ^196^ ^]^ reported the synthesis of thermosensitive polymers with gold NPs so that gold NPs bound to the polymer become sensitive to temperature. **Figure** **6**a presents the images of a thermoresponsive polymer that contains gold NPs and the results of optical properties according to temperature. Lewandowski et al.^[^ ^197^ ^]^ demonstrated that utilizing silver NPs coated with a thermally responsive organic material, it is possible to create a metamaterial that exhibits reversible switching characteristics. The studied material displays dynamic self‐assembly, which arises from temperature‐dependent alterations in the shape of the organic coating, leading to a modifiable spatial distribution of the silver NPs. Consequently, this significantly impacts the optical properties of the entire material. Lee et al.^[^ ^198^ ^]^ reported the development of plasmonic metamolecules that possess tunable optical magnetism. These metamolecules were produced by decorating gold or silver nanobeads onto a thermoresponsive PPA hydrogel sphere, which results in uniform core satellite‐type assembly structures that exhibit a consistent interbead distance and strong interparticle coupling.

Beyond simple polymer structures, liquid crystalline matrix‐based assemblies can lead to chiral properties for plasmonic NPs. Grzelak et al.^[^ ^195^ ^]^ fabricated plasmonic circular dichroism (PCD) thin films using liquid crystals, chiral dopants, and gold NPs and designed reversible on/off switching of chiroptical response by changing the substrate temperature (Figure 6b). The researchers demonstrated the reversibility of the material, allowing active control over PCD properties in thin‐film configurations by changing the temperature of the surrounding environment as external stimuli. Even if they used achiral gold NPs, active plasmonic properties and mechanical tunability could be achieved with chiral arrangements of coupled NPs in liquid crystals assembling.

**Figure 7:**
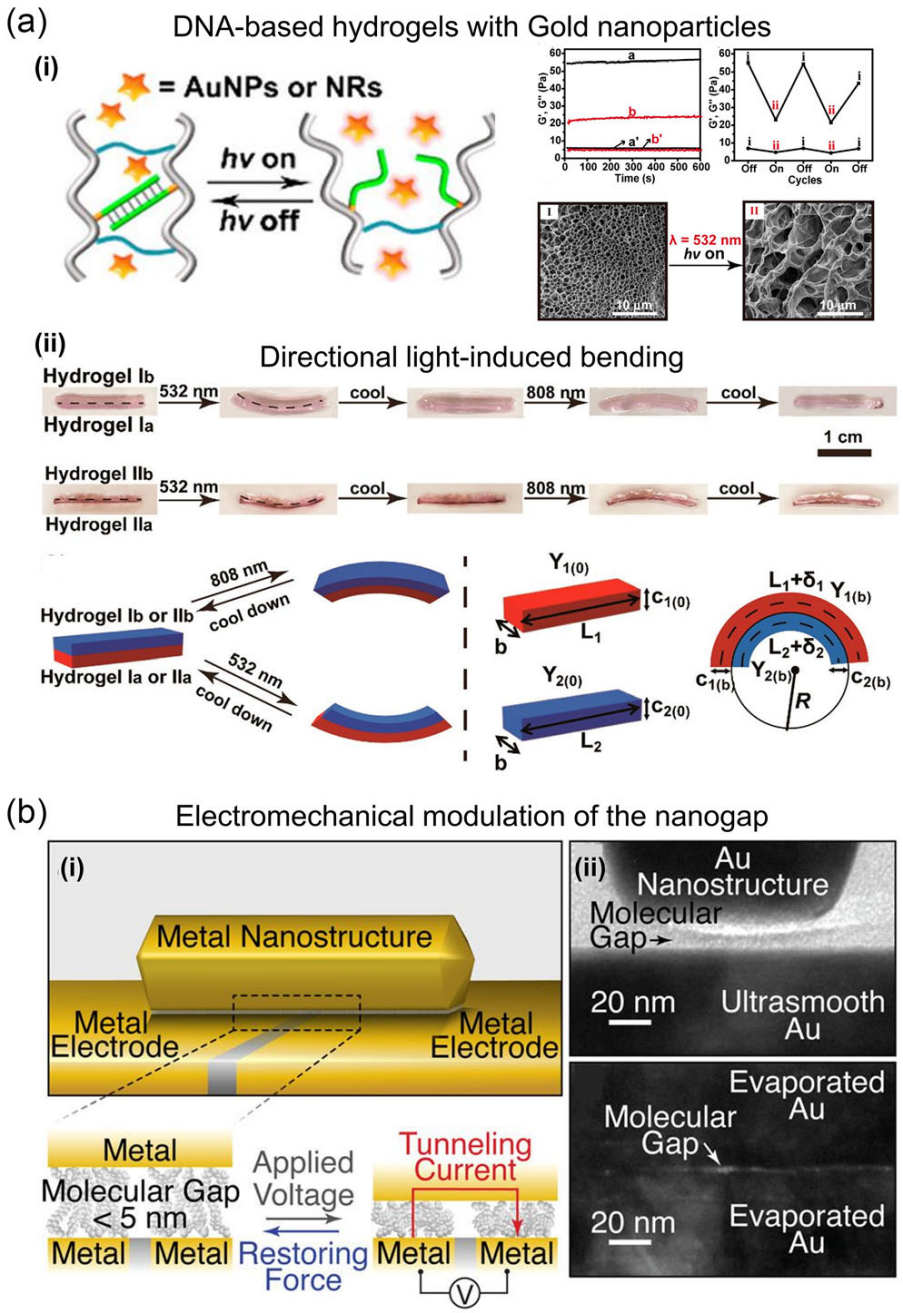
Chemical‐assisted photothermal and electrical switching. a) Reversible thermoplasmonic light‐induced bending. i) Left: schematic illustration of light‐induced thermoresponsive stiffness control of crosslinked hydrogels. Upper right: Rheometric characterization and switchable on/off stiffness control of crosslinked hydrogel in the dark and upon irradiation at 532 nm. Lower right: SEM images of crosslinked hydrogel in the dark and after irradiation at 532 nm. ii) Upper: Reversible thermoplasmonic light‐induced bending of the crosslinked hydrogels bilayer composites. Lower: Schematic representation of the geometrical parameters used to calculate the curvatures of the respective irradiated bilayer composites. a) Reproduced with permission.^[^ ^200^ ^]^ Copyright 2019, American Chemical Society. b) Electrically active molecular junction. i) Schematic of a device with ultrasmooth bottom contacts connected by a NR and a self‐assembled molecular layer. The mechanical mobility of the top contact allows for gap reconfiguration during actuation. ii) TEM cross‐sectional image (upper) of a uniform molecular junction formed between an ultrasmooth gold thin film and an atomically flat facet of a gold NR. TEM cross‐sectional image (lower) of a nonuniform molecular junction formed between thermally evaporated gold contacts. b) Reproduced with permission.^[^ ^201^ ^]^ Copyright 2021, American Chemical Society.

In contrast to the use of temperature change in the surrounding medium, induction of thermal effect via irradiating light or applying AC voltage was proposed,^[^ 199, 200, 201 ^]^ for example, with light irradiation to induce a photothermal effect on DNA‐conjugated plasmonic NPs. Wang et al.^[^ ^200^ ^]^ incorporated plasmonic nanostructures into hydrogels to create versatile materials with controllable mechanical properties. Gold NPs and gold NRs were loaded into the crosslinked hydrogel and the stiffness was controlled using thermoplasmonic heating. When irradiated by either 532 nm (for loading with gold NPs) or 808 nm light (gold NRs), hydrogels underwent thermoplasmonic heating, which caused dehybridization of DNA duplexes and resulted in lower stiffness. By turning the light source on or off, the stiffness of hydrogels was altered from a low to a high state. This reversible management of mechanical properties allowed the development of the directional light‐induced bending (**Figure** **7**a), as well as the tailoring of thermoplasmonic switchable drug release. Niroui et al.^[^ ^201^ ^]^ designed active molecular junctions with controllable self‐assembled molecular nanogaps, the size of which is tuned by applying electrostatic forces. When a top contact responds to applied forces, it attracts toward the bottom, resulting in reduced molecular nanogap width (Figure 7b). The decreased molecular gap size can increase the tunneling current exponentially. This electromechanical modulation of the sub‐5‐nm nanogap provides an additional level of control for the active engineering of current conduction.

#### 3.2.3 Electromagnetic‐Field‐Induced Structure‐Tunable Switching

**Figure 8:**
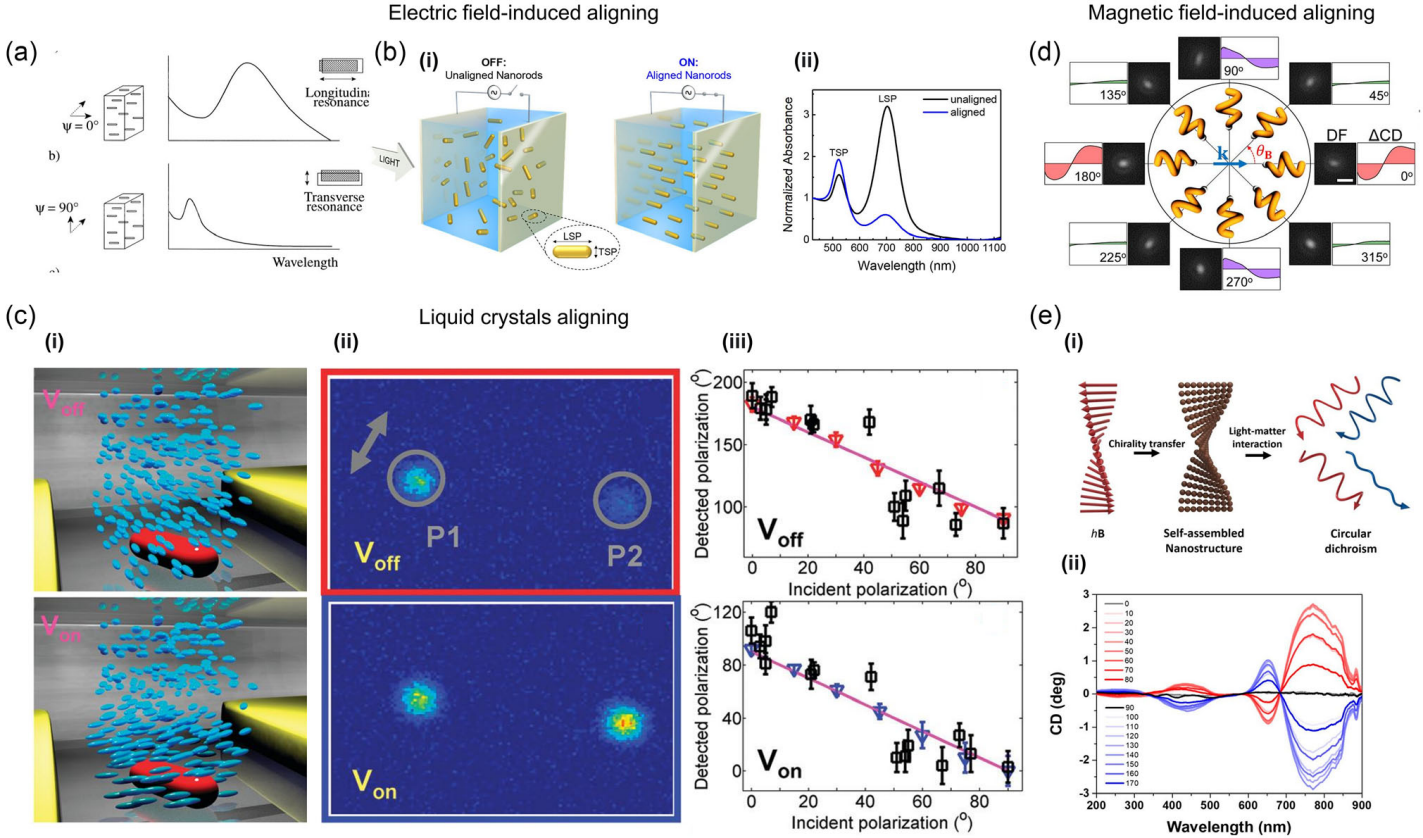
Electromagnetic‐field‐induced NP aligning. a) The absorption of light by colloidal dispersions of gold NRs was represented schematically. The absorbance spectrum for aligned gold rods was shown to exhibit the longitudinal resonance when parallel polarized light was used (upper) and the transverse resonance when perpendicular polarized light was used (lower). Reproduced with permission.^[^ ^202^ ^]^ Copyright 1999, American Chemical Society. b) Electric‐field‐induced gold NR composition alignment. i) A schematic illustration of the dynamic plasmonic pixel is provided in the schematic diagram when an electric field is applied to it, both in an unaligned state (0 V μm^−1^) and in an aligned state (6 V μm^−1^). ii) The extinction spectra of a suspension of polystyrene‐thiol‐coated gold NRs in toluene were measured while the electric field was off (black) and on (blue). b) Reproduced with permission.^[^ ^203^ ^]^ Copyright 2019, American Chemical Society. c) Electric‐field‐induced twisted nematic alignment. i) Schematic illustration of an electric‐field‐induced shift from a uniform to a twisted nematic phase. ii) Polarized dark‐field scattering images in the absence and presence of an electric field. iii) The observed light polarization varied with the NR orientation for *V*_off_ and *V*_on_ (represented by black symbols). c) Reproduced with permission.^[^ ^126^ ^]^ Copyright 2011, American Chemical Society. d) Schematic illustration and dark‐field images of magnetically driven chiroptical switching. Reproduced with permission.^[^ ^204^ ^]^ Copyright 2016, American Chemical Society. e) Chiroptical properties of magnetoplasmonic NPs under helical magnetic field (hB). i) Schematic illustration of hB‐induced chiroptical property. ii) The circular dichroism spectra of magnetoplasmonic superstructures that assembled with different values of *θ* (0°≤ *θ* < 180°). e) Reproduced with permission.^[^ ^205^ ^]^ Copyright 2020, American Chemical Society.

The response of NPs to electromagnetic fields has been widely exploited to mechanically convert plasmonic characteristics with various methods. One of the popular methods is to manipulate the alignment of anisotropic plasmonic materials by exerting an electric field. In this method, the absorption of materials containing plasmonic particles as electro‐optical particles, such as colloidal dispersions of metal NPs, can be directly manipulated. Zande et al.^[^ ^202^ ^]^ reported electrical field effects on aligning colloidal dispersion of gold NRs with various aspect ratios. In this study, the optical properties were analyzed theoretically according to the orientation of the aligned gold NRs with respect to the applied electric field. Experimental demonstration was conducted by measuring absorbance with aqueous dispersion of gold NRs according to the aspect ratios of NRs and electrical voltages. **Figure** **8**a shows a schematic representation of the absorbance spectra according to the alignment state of the gold NRs. Greybush et al.^[^ ^203^ ^]^ demonstrated that dynamic plasmonic pixels can enable the manipulation of light in terms of its spectral, spatial, and temproal aspects. This is achieved by aligning plasmonic NRs in organic suspensions induced by electric fields (Figure 8b). They customized the geometry and composition of the NRs, using composition (Au and Au@Ag), and were able to achieve light modulation across a substantial range of infrared and visible spectra. This rapid and reversible alignment of the NRs, occurring within ≈30 μs, resulted in noticeable changes in color, as evidenced by shifts in both chromaticity and luminance. In addition to directly aligning plasmonic NPs, a way to indirectly switch the plasmonic properties of NRs using liquid crystals, which are widely used as anisotropic materials and enable electro‐optical reactions, was also found. Khatua et al.^[^ ^126^ ^]^ reported active plasmonic modulation of gold NRs between electrodes using twisted nematic phase by applying an electric field to liquid crystals. Polarized dark‐field images were presented according to the alignment state of anisotropic liquid crystals to demonstrate plasmonic switching of gold NRs (Figure 8c).

Similarly, magnetic fields have been exploited to switch plasmonic characteristics. In general, magnetoplasmonic materials have been synthesized by incorporating magnetic materials into plasmonic particles to allow actuation by magnetic force for active plasmonic behavior. Jeong et al.^[^ ^204^ ^]^ reported that dynamic plasmonic NPs possess the potential to function as mechanical sensors that can selectively investigate the rheological characteristics of fluids at the nanoscale and microscopic levels. They synthesized chiral magnetoplasmonic nanocolloids, which can be stimulated by an external magnetic field, facilitating rapid and direct modulation of their unique optical response. Figure 8d presents schematic illustrations of active magnetoplasmonic switching with magnetically driven circular dichroism, which can be applied to active nanorheology. Jeong et al.^[^ ^205^ ^]^ reported a method to construct helical superstructures with chiroptical properties using a mimetic helical magnetic field (hB)‐assisted self‐assembly of magnetoplasmonic Ag@Fe_3_O_4_ core–shell NPs and plasmonic silver NPs. The helical magnetic flux guides plasmonic silver NPs. The chirality and circular dichroism of the assembled structures were tuned in real time. The hB modulates the chirality of the assembled structures at the millisecond level, which is significantly faster than other template‐assisted methods. The circular dichroism peak can be reconfigured by controlling the silver core size and magnetic flux density. This method provides a way of controlling the polarization state of light and combines various domains, including plasmonics, magnetic self‐assembly, colloidal science, liquid crystals, and chirality. Figure 8e presents schematic images of a helical magnetic field and shows the chiroptical properties of magnetoplasmonic NPs.

### 3.3 Active Material‐Based Switching

Active material‐based switching is another approach involving external stimuli to modulate material properties of the plasmonic nanostructure or surrounding materials. The approach is based on the variation of the material property itself in contrast to modulating the spatial relationship between plasmonic nanostructures such as the alignment and period. Active material‐based switching can be achieved through diverse mechanisms, such as phase transition and electrical‐ and optical‐stimuli‐responsive effects.

#### 3.3.1 Phase‐Transition Material‐Based Switching

**Figure 9:**
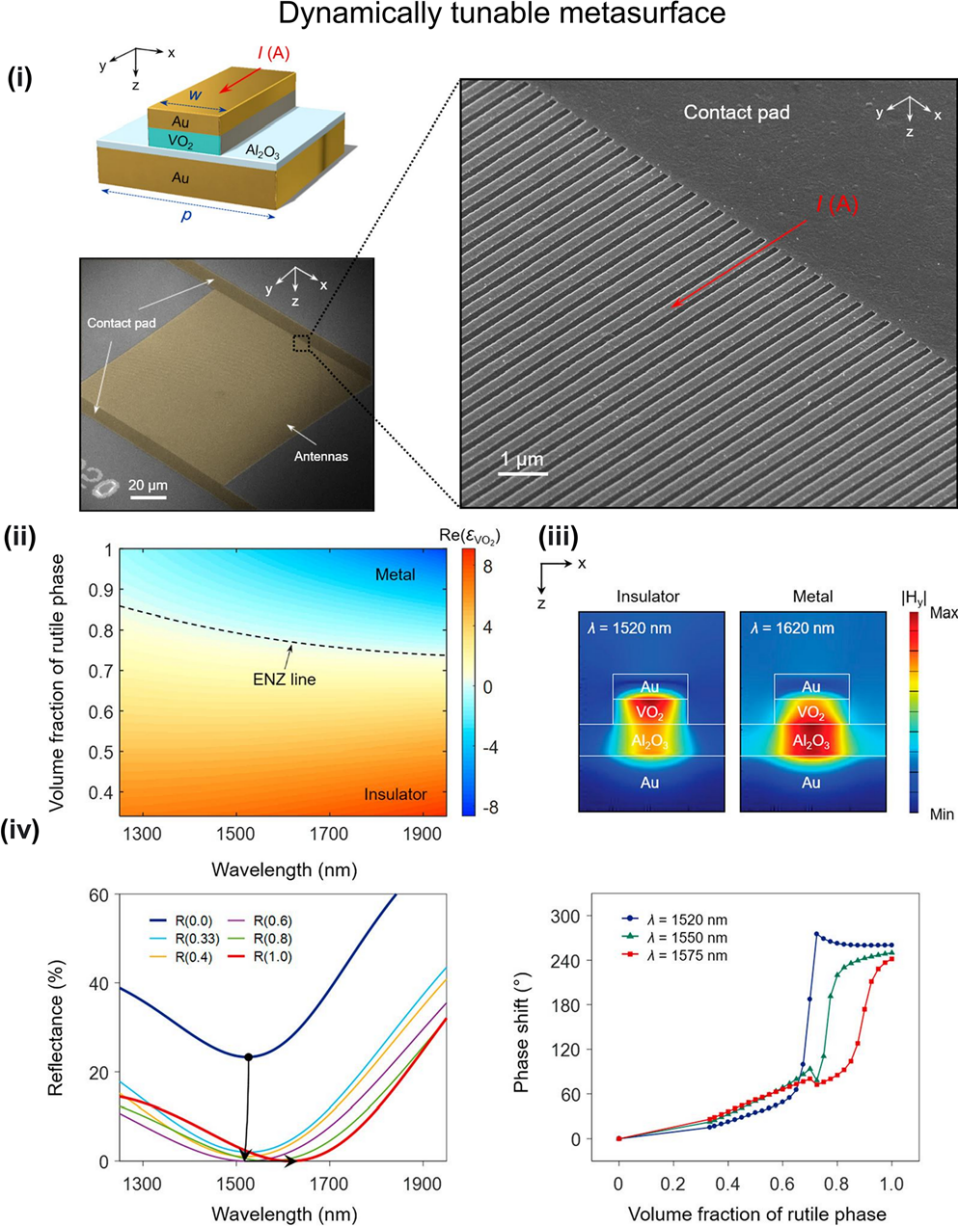
Phase‐transition material‐based switching. Dynamic tunable reflectarray metasurface based on VO_2_ as active material. i) Schematic illustration of electrically tunable VO_2_ metasurface and its SEM images. Simulation results of the electrically tunable metasurface structure. ii) Relationship between the real part of the dielectric permittivity of VO_2_ and the volume fraction of rutile phase. The intermediate dielectric permittivity is evaluated by implementing the Bruggeman effective medium model. The region where the real part of the dielectric permittivity of VO_2_ equals zero is represented by a dashed curve, indicating an abrupt change in VO_2_ optical characteristics. iii) Magnetic field magnitude in the unit cell of metasurface, with VO_2_ in the dielectric phase (left), and metallic phase (right) under normal incidence at each resonance wavelength. iv) The spectral reflectance of the VO_2_ layer is according to volume fractions of the rutile phase. Additionally, the modulation of phase is investigated as a function of the rutile phase volume fraction in the VO_2_ layer for three wavelengths. i–iv) Reproduced with permission.^[^ ^140^ ^]^ Copyright 2019, American Chemical Society.

Phase transition material offers components to implement active plasmonic switching by controlling the phase of materials such as in metallic or crystalline state. Kruger et al.^[^ ^206^ ^]^ reported a design of plasmonic switching that utilizes the hybridization of single‐interface SPP and thin‐film modes of transition metal oxide material, specifically VO_2_. Localized heaters were incorporated into the design and used to initiate VO_2_ transition. Kim et al.^[^ ^140^ ^]^ reported a reflectarray metasurface that is dynamically tunable, continuously modifying the reflected light phase in the near‐infrared wavelength range. This is achieved by dynamically controlling VO_2_ phase transition from the semiconducting state to the semimetallic state with electronic heating. The proposed method involves integrating an active layer of VO_2_ into the dielectric gap of antenna elements in a reflectarray metasurface. When metallic patch antennas are resistively heated, the VO_2_ active layer undergoes a transition from insulator to metal, which disturbs the magnetic dipole resonance that the metasurface supports. **Figure** **9** presents a schematic illustration and SEM images of the device, showcasing its working principle that utilizes the VO_2_ layer as a phase transition material. Chen et al.^[^ ^207^ ^]^ reported on the tunability of lattice resonance in a hybrid plasmonic crystal, where a 20 nm‐thick layer of the phase‐change material Ge_2_Sb_2_Te_5_ (GST) was sandwiched between a gold nanodisk array and a quartz substrate. Their work showcases the effectiveness and convenience of using GST to adjust resonance in the near‐infrared range. Yin et al.^[^ ^208^ ^]^ reported implementation of a bifocal zoom lens as well as beam switching with active plasmonic metasurface that adopted GST as phase‐change material. This is achieved by actively controlling the phase change from amorphous to crystalline states using temperature control.

#### 3.3.2 Material‐Based Switching in Response to an Electrical Stimulus

Response to electrical stimuli can be implemented in numerous ways, for example, by exerting electrostatic forces or applying electric field effects. In addition to the demonstration of the ability of electrostatic forces to mechanically actuate plasmonic structures (Section 3.2.1), here we describe an approach that utilizes electrostatic forces as a memristive device to modulate material properties. Emboras et al.^[^ ^209^ ^]^ reported an electrically controlled plasmonic switch that operates at the atomic scale. This switching method allows rapid and consistent switching through the relocation of a single atom or, in some cases, a few atoms, within a plasmonic cavity.

**Figure 10:**
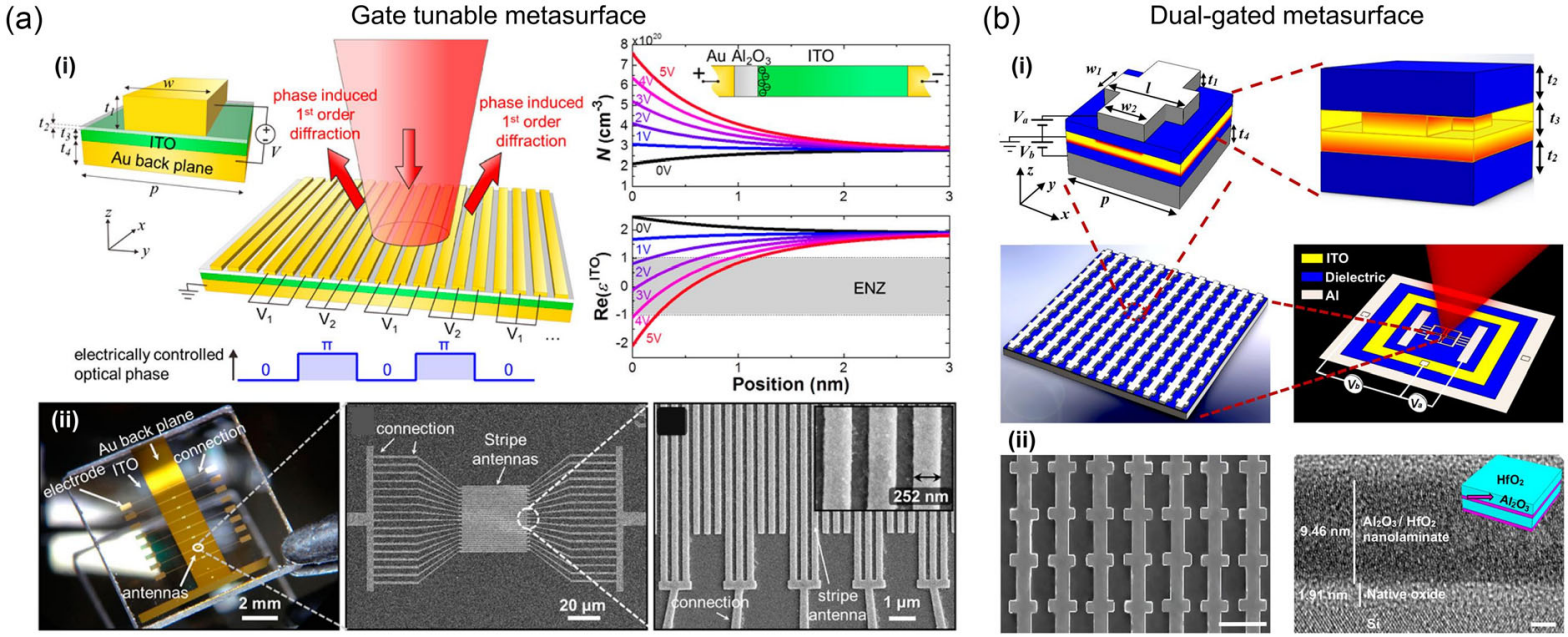
Material‐based switching in response to electrical stimuli. a) Gate‐tunable reflectarray metasurface. i) Schematic of the gate‐tunable metasurface and its operation principle. The metasurface is based on MOS field‐effect dynamics which enables to utilize tuning of an electron accumulation in the ITO side of the region of the interface between Al_2_O_3_/ITO. ii) Photographic image of the device consists of a gate‐tunable metasurface and zoomed SEM images. a) Reproduced with permission.^[^ ^138^ ^]^ Copyright 2016, American Chemical Society. b) Dual‐gated tunable reflectarray metasurface. i) Schematic of the illustration the dual‐gated metasurface and its geometrical details. ii) SEM image (left) of the dual‐gated metasurface (scale bar: 500 nm). TEM image (right) of an Al_2_O_3_/HfO_2_ nanolaminate, which functions as a gate dielectric in dual‐gated metasurface (scale bar: 2 nm). b) Reproduced with permission.^[^ ^210^ ^]^ Copyright 2018, American Chemical Society.

As a way to apply electric field effects, field‐effect tunable materials allow the development of dynamically tunable metasurfaces that can be actively controlled to change both the phase and amplitude of the reflected light. ITO is a commonly used conducting oxide material with applications for the manipulation of charge distribution through electric field effects. Huang et al.^[^ ^138^ ^]^ reported a metasurface that has the ability to dynamically control the reflected plane wave through gate tunability. This tunability is achieved by modulating the complex refractive index of the conducting oxide layers incorporated into the metasurface antenna elements arranged in a reflectarray geometry. **Figure** **10**a shows the schematic diagram and operating principle of the device in which ITO is used as the electrical‐stimuli‐responsive material and images of an actual gate‐tunable conductive oxide device. Shirmanesh et al.^[^ ^210^ ^]^ reported an architecture for dual‐gated reflectarray metasurface that offers a wider range of phase tunability. They investigated the interactions between light and matter within the metasurface elements, which incorporate two independent voltage‐controlled metal–oxide–semiconductor (MOS) field‐effect channels connected in series to form a single element. The use of ITO as the active stimuli‐responsive material and a composite hafnia/alumina gate dielectric allows wide phase tunability. Figure 10b presents a schematic illustration and SEM images of a dual gate‐tunable device, respectively.

#### 3.3.3 Material‐Based Switching in Response to an Optical Stimulus

Aside from electrical stimuli, which provide electrical gating as a method of modulating the free carrier density of conductive materials, an emerging methodology is to use optical stimuli for photocarrier excitation. Although a limited range of charge density and index changes in accumulation or depletion layers remains as a significant obstacle, recent works have shown that photocarrier excitation in transparent conductive oxides such as ITO and aluminum‐doped zinc oxide offers an effective solution.^[^ 42, 139, 211, 212, 213, 214, 215 ^]^ Alam et al.^[^ ^211^ ^]^ revealed that an ITO film can undergo significant optical modulation, with a remarkable 170% change in its refractive index and a fast recovery time of ≈360 fs. This index modulation can be utilized to significantly alter the spectral scattering properties of plasmonic antennas that are positioned on top of the ITO layer.^[^ 213, 215 ^]^ One of the applications is an ultrafast all‐optical switch based on the enhanced nonlinear absorption of corrugated ITO thin films, which overcomes the limitations of narrowband, polarization‐dependent, and angle‐dependent characteristics of ENZ materials.^[^ ^215^ ^]^ This approach provides a highly nonlinear saturable absorption coefficient and ultrafast switching time of 350 fs, demonstrating its potential as an ENZ ultrafast all‐optical switching material platform. The optical stimuli may replace thermal and electronic triggers even in phase‐change materials. Michel et al.^[^ ^216^ ^]^ demonstrated reversible modulation of infrared antenna array resonances using femtosecond‐laser‐induced nonvolatile structural state changes in GST. The reversible optical modulation system allows single‐antenna array addressing independent switching without thermal barriers.

## 4 Applications of Switching Plasmonics and Engineered Nanostructures

With the advancement in switching plasmonics, the controllability of optical signals is improved over that of nonswitching counterparts. The switching platform has evolved to enable multifaceted analysis by enabling a single device or system to have multiple functions or to acquire diverse signals. This section provides a brief overview of recent applications with switchable plasmonic nanostructures, particularly in biosensing and imaging, energy and light harvesting, and dynamic optical components in color displays.

### 4.1 Applications in Biomedical Engineering

#### 4.1.1 Biosensing Applications

**Figure 11:**
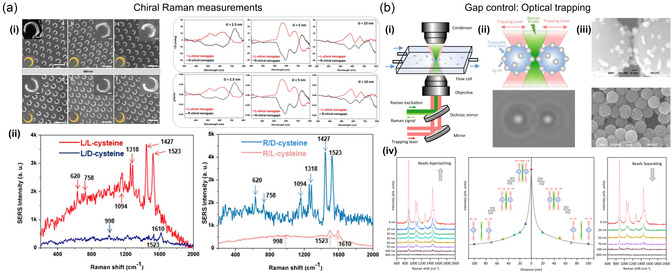
SERS applications. a) Plasmonic chiral nanogaps. i) SEM images (left) of l‐chiral and R‐chiral nanogaps with *G* = 2.5, 5, and 10 nm, respectively (scale bar: 1 μm). Zoomed‐in SEM images of the corresponding chiral nanogaps are shown in the insets (scale bar: 100 nm). Circular dichroism spectra (right) and *g*‐factor of chiral nanogaps with the different widths of *G* = 2.5, 5, and 10 nm, respectively. ii) On the L‐chiral nanogaps with *G* = 5 nm, l‐cysteine (red curve), and d‐cysteine (blue curve), Raman spectra were captured in left plot, and the Raman spectra of d‐ and l‐cysteine recorded on R‐chiral nanogaps with *G* = 5 nm (pink curve and cyan‐blue curve, respectively). a) Reproduced with permission.^[^ ^220^ ^]^ Copyright 2021, American Chemical Society. b) Optical‐tweezers‐controlled hotspot for sensitive SERS. i) Schematic representation of the microfluidic optical tweezers‐coupled Raman spectroscopic platform. ii) Upper: Schematic illustration of two trapping laser beams (red) to manipulate two silver‐NP‐coated beads and one Raman probe beam (green) to detect signals from the gap between the two silver‐NP‐coated beads. Lower: Two trapped silver‐NP‐coated beads are shown in the real‐time camera image from the microscopy. iii) SEM image of the gap between two silver‐NP‐coated beads (scale bar: 0.1 μm). Zoomed‐in SEM image of silver‐NP‐coated beads to show the uniform silver‐NP‐coated (scale bar: 1 μm). iv) Left: SERS spectra of 1% ethanol aqueous solution with 1 s acquisition time when the two silver‐NP‐coated beads approached. Center: Intensity of the ethanol characteristic peak at 1458 cm^−1^ as a function of the distance between the two silver‐NP‐coated beads from beads approaching to beads separating. The reversible bead positions are illustrated as inset. Right: SERS spectra of 1% ethanol aqueous solution with 1 s acquisition time when the two silver‐NP‐coated beads separated. b) Reproduced under the terms of the CC‐BY Creative Commons Attribution 4.0 International license (https://creativecommons.org/licenses/by/4.0).^[^ ^222^ ^]^ Copyright 2021, The Authors, published by Springer Nature.

SERS is a powerful analytical technique that enhances the Raman signal of molecules adsorbed onto metallic surfaces through the use of plasmonic nanostructures and is increasingly recognized as a valuable tool in biomedical applications.^[^ 92, 217 ^]^ Recent studies reported chirality in plasmonic nanostructures for applications in chiral sensing, circular dichroism spectroscopy, and asymmetric catalysis.^[^ 218, 219, 220, 221 ^]^ Switching the chirality of plasmonic nanostructures becomes an increasingly important area of research, as it enables the development of new types of chiral sensors and devices with tunable optical properties. Zhang et al.^[^ ^220^ ^]^ selectively excited chiral Raman signals using chiral nanogaps that are smaller than 10 nm and possess tailored chiroptical responses. The researchers fabricated these nanogaps using a cooperation of nanoskiving method and glancing angle deposition. By breaking the mirror symmetry of the nanostructure, they induced robust plasmonic coupling in the tips and gaps, resulting in the occurrence of circular dichroism activity. Chiral nanogaps that are receptive to SERS enable the chiral‐selective engagement with both l‐and d‐cysteine molecules, leading to distinct SERS outcomes. As shown in **Figure** **11**a, l‐chiral nanogaps/l‐cysteine exhibit more pronounced Raman intensities compared to l‐chiral nanogaps/d‐cysteine owing to the interplay between molecular conformation of l‐cysteine and oscillating electron cloud. In a similar fashion, R‐chiral nanogaps/d‐cysteine exhibit more robust Raman signals compared to R‐chiral nanogaps/l‐cysteine. It suggests that specific cysteine chiral‐isomers can be selectively recognized by regular Raman spectroscopy, without requiring chiral labels or incident light with circular polarization.

As a different approach to sensitive and reproducible SERS detection, Figure 11b shows the potential of nanoscale structure‐tunable plasmonic switching for dynamic control of SERS.^[^ ^222^ ^]^ Dai et al. adjusted the gap distance of silica beads coated with silver NPs by optical trapping. Based on SERS detection with an optical tweezer, the intensity of ethanol characteristic peaks was enhanced as the silica beads approached each other. This strategy allows precise control of a plasmonic hot spot between two trapped micrometer‐sized silver NP‐coated beads, improving SERS efficiency and reproducibility in aqueous detection, while also offering subnanometer spatial resolution and force sensitivity on the order of sub‐pN for monitoring light–matter interactions.

#### 4.1.2 Biomedical Imaging Applications

Imaging applications of plasmonic switching can be broadly divided into two categories: SPR microscopy (SPRM)^[^ 3, 223, 224, 225, 226 ^]^ and fluorescence‐enhanced imaging.^[^ 150, 227, 228, 229, 230 ^]^ For imaging, switchable properties of plasmonics are important since they open up to acquire signals with multiple characteristics. Diverse signals can be used in many ways such as improving image contrast, reducing noise, and thereby providing more accurate information about the sample. This section addresses the performance‐enhancing role of plasmonic switching for two representative plasmon‐enhanced imaging techniques.

**Figure 12:**
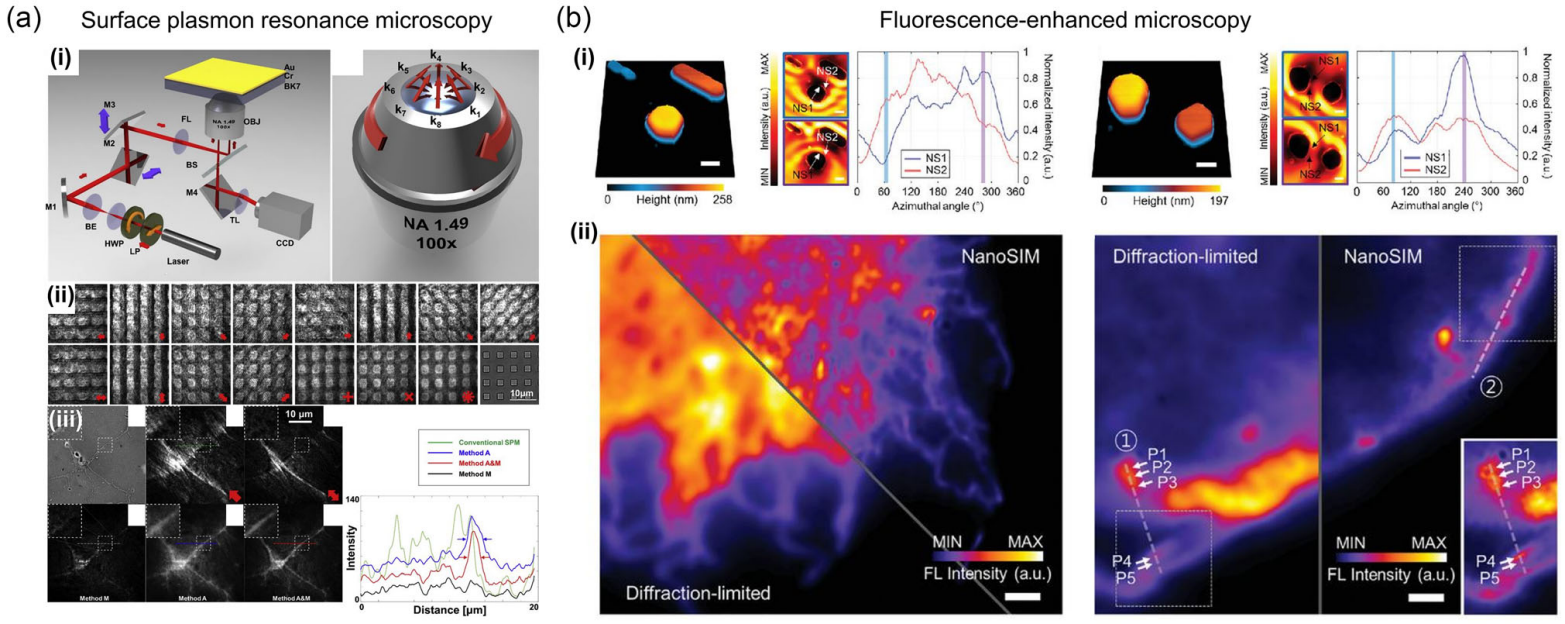
Biomedical imaging applications. a) Multichannel spatially switched SPRM. i) Optical setup and conceptual illustration of switched light illumination in eight‐channel incidence. ii) Images of reference square array by conventional SPRM and spatially switched SPRM. The array period and square width are 5 and 2 μm, respectively. iii) Images of a cortical neuron by eight‐channel spatially switched SPRM. Upper: Bright‐field image of the neurons, conventional SPRM, and two‐channel spatially switched SPRM image of the neuron are shown. The red arrow marks the direction of light incidence. Lower: Eight‐channel minimum filtering, averaging, and minimum‐filtered two‐channel averaging are demonstrated. The right plot demonstrates intensity distribution across neuronal axons with respect to the image reconstruction methods compared to conventional SPRM. a) Reproduced with permission.^[^ ^226^ ^]^ Copyright 2019, Elsevier. b) Nanospeckle illumination microscopy. i) Two different gold nanoisland region (left) AFM images of a magnified nanoisland substrate (scale bar: 300 and 200 nm, respectively). Localized near‐field distributions (right) on the corresponding region of the gold nanoisland, representing the blue line and violet line of the corresponding normalized intensity profile and the normalized intensity profile represents the values at the two selected points in near‐field distributions. ii) Fluorescence images of ganglioside distribution on the HeLa cell membrane with diffraction‐limited and NanoSIM images (scale bar: 1 μm). b) Reproduced with permission.^[^ ^230^ ^]^ Copyright 2021, Wiley‐VCH.

Son et al.^[^ ^226^ ^]^ investigated multichannel spatially switched SPRM to overcome the limitations of conventional SPRM due to severe SPR scattering artifacts (**Figure** **12**a). They utilized eight‐channel light incidence directions for robust imaging performance of neuron cells, resulting in a significant enhancement in contrast by over three times compared to conventional SPRM with much reduced scattering artifacts of SPR. By switching the light incidence in multiple directions, this technique allows for robust and consistent imaging of complex patterns and shapes.

On the other hand, Yoo et al.^[^ ^230^ ^]^ introduced the use of disordered gold nanoisland structures for fluorescence‐enhanced microscopy as nanospeckle illumination microscopy (NanoSIM). This approach controls LSPR effects based on changes in the azimuthal direction of light propagation. As shown in Figure 12b, gold nanoislands generate diverse near‐field distributions that excite an arbitrary number of fluorescence‐enhanced images to achieve super‐resolution. The experimental results show that NanoSIM with 180 images of HeLa cells improve spatial resolution by more than three times over diffraction‐limited imaging. Compared to many studies of plasmon‐enhanced microscopy that produced super‐resolution with sophisticated lithographic techniques, NanoSIM emphasizes that super‐resolution can be achieved by switching random plasmonic nanostructures patterned without lithography.

### 4.2 Energy Applications

In this section, we discuss energy applications based on plasmonics, in particular, energy harvesting and plasmonic heating as nanoscale heat sources. Plasmonic nanostructures may convert light energy into chemical and electrical energy to produce enhanced catalytic reaction,^[^ 231, 232, 233 ^]^ thermal desalination,^[^ 234, 235, 236 ^]^ and renewable energy.^[^ 237, 238 ^]^ In addition, plasmonic materials under intense light absorption may act as nanoscale heat sources and be applied to plasmonic photothermal therapy (PTT),^[^ 239, 240, 241 ^]^ cell engineering,^[^ 242, 243 ^]^ and plasmon‐assisted optofluidics.^[^ 244, 245, 246 ^]^ More details of plasmon‐assisted energy applications for which plasmonic switching can be useful are described in what follows.

#### 4.2.1 Energy Harvesting

**Figure 13:**
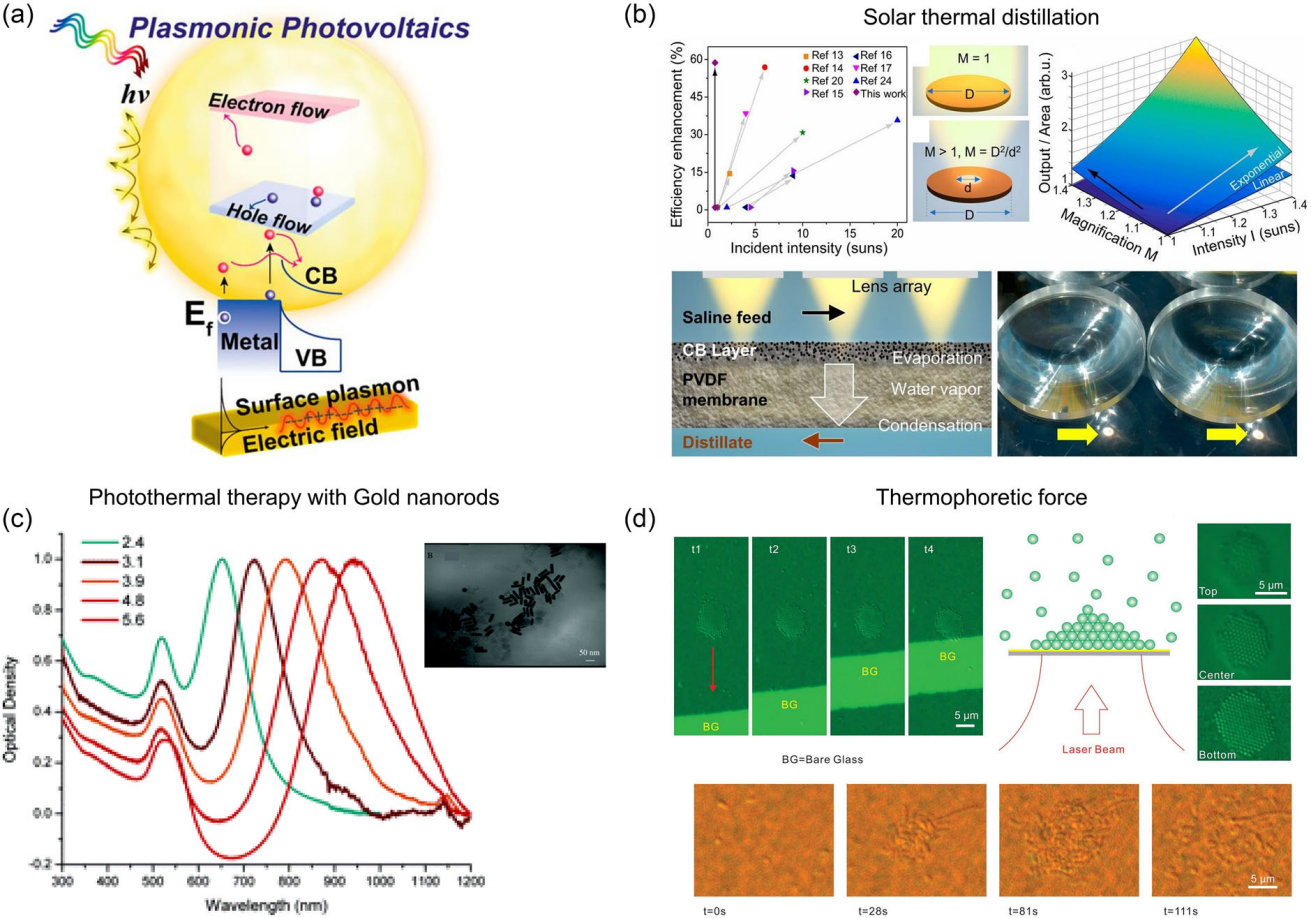
Energy harvesting and nanoscale heat source applications. a) Schematic of plasmonic photovoltaics. Reproduced with permission.^[^ ^357^ ^]^ Copyright 2016, American Chemical Society. b) Efficiency enhancement and nonlinear optical process on carbon black‐coated polyvinylidene difluoride membrane to purify water by solar thermal desalination. Reproduced with permission.^[^ ^236^ ^]^ Copyright 2019, National Academy of Sciences, USA c) Optical spectrum of gold NR with regard to aspect ratio and TEM image used in PTT. Reproduced with permission.^[^ ^284^ ^]^ Copyright 2006, American Chemical Society. d) Manipulation of polystyrene beads and *Escherichia coli* using plasmon‐assisted thermophoretic force by plasmonic nanoisland. Reproduced under the terms of the CC‐BY Creative Commons Attribution 4.0 International license (https://creativecommons.org/licenses/by/4.0).^[^ ^245^ ^]^ Copyright 2015, The Authors, published by Springer Nature.

Energy harvesting based on plasmonics often takes advantage of optical properties of plasmonic NPs to generate electric power or perform energy‐related tasks.^[^ 12, 247 ^]^ The principles behind this process are photovoltaic and photothermoelectric process. When illuminated with light at specific wavelengths, plasmonic nanostructures can generate intense local electric fields and be harnessed to produce energy. **Figure** **13**a presents a schematic of plasmon‐assisted photovoltaic response which may be accelerated by LSPR and charging effect on plasmonic materials.^[^ ^248^ ^]^ One of the applications of plasmonic energy harvesting is in the development of solar cells that are more efficient than traditional photovoltaic cells. Traditional photovoltaic cells tend to have limited performance due to low absorption efficiency and poor response to light at certain wavelengths. In comparison, plasmonic nanostructures may overcome these limitations by manipulating absorption spectrum to improve optical response of solar cells while employing localized electric field to separate electrons and holes in the active layer of a solar cell to generate a voltage difference that can produce electrical energy.^[^ 249, 250 ^]^ To manipulate absorption spectrum of solar cells and trap electromagnetic fields on the solar cell layer for improved efficiency, various plasmonic nanostructures have been proposed, such as NPs,^[^ 251, 252, 253 ^]^ nanostars,^[^ ^254^ ^]^ NRs,^[^ 255, 256 ^]^ nanoprisms,^[^ ^257^ ^]^ nanocones,^[^ ^258^ ^]^ nanopillars,^[^ ^259^ ^]^ nanowells,^[^ ^260^ ^]^ and nanoholes.^[^ ^261^ ^]^

Meanwhile, absorbed light energy in plasmonic material can be converted into heat and generate electricity through photothermoelectric processes. In thermoelectric energy harvesting, the temperature difference between a plasmonic NP and its surrounding is used to produce an electric current. In contrast, heat generated by the plasmonic NP in thermophotovoltaic energy harvesting may excite electrons in a nearby photovoltaic cell and then generate an electric current. Several studies have attempted manipulation of plasmonic characteristics to improve solar power conversion efficiency, for example, using metamaterials,^[^ 262, 263 ^]^ near‐field power transfer/thermal radiation,^[^ 264, 265 ^]^ and plasmonic absorbers.^[^ 266, 267 ^]^

Interestingly, plasmonic energy harvesting has been expanded to control the catalytic process due to an ability of plasmonic materials to enhance light absorption and energy conversion.^[^ ^268^ ^]^ Photocatalyst has an advantage of reducing activation energy of chemical reactions and is easy to pause reaction process when incident light is blocked. Because LSPR contributes to strong light absorption and excitation of hot carriers that help reduce the activation barrier of chemical reactions,^[^ 268, 269 ^]^ many groups studied plasmonic photocatalytic systems based on excitation of hot carriers and plasmonic heating to enable high‐efficiency photocatalysis.^[^ 270, 271, 272, 273, 274, 275, 276 ^]^ For example, Halas et al.^[^ ^271^ ^]^ reported the origin of plasmonic photocatalysis that compares with the relation between thermal and electronic excitation. In a recent review, Mateo and co‐workers presented photothermal catalysis which is based on several mechanisms, such as LSPR and photothermal effect.^[^ ^277^ ^]^ They raised several limitations of photothermal catalysts: difficult usage of a continuous flow system, need of understanding dominant pathways of the reaction mechanism, scarcity of materials, and issues of long‐term stability. Plasmon‐mediated solar desalination was reported in a number of research groups as a variant of plasmonic catalysis.^[^ 234, 235, 236 ^]^ As shown in Figure 13b, Dongare et al.^[^ ^236^ ^]^ reported nanophotonics‐enabled solar membrane distillation using carbon black NPs in polyvinyl alcohol to enhance solar energy efficiency up to 53.8% and acquired purified water of ≈4 L day^−1^.

#### 4.2.2 Nanoscale Heat Source

PTT is a therapeutic strategy that utilizes light energy to generate localized heat in a targeted tissue or cell population, thereby induce thermal damage or destruction of targeted cells or tissues. This approach involves the use of light‐absorbing agents, such as plasmonic NPs, which convert absorbed light into heat via plasmonic heating. In PTT, the light‐absorbing agents are delivered to the target area, either directly or through carriers that include ligands such as antibodies, nucleic acid aptamers, and peptides.^[^ ^278^ ^]^ The light‐absorbing agents, for example, plasmonic NPs can be directly delivered to the target area through leaky tumor vasculature which is more permeable than normal tissue. Lymphedema of tumor induces light‐absorbing agents to be more distributed in tumor than in normal tissues.^[^ 279, 280 ^]^ For clinical application, the incident light wavelength of PTT should be carefully selected in the near‐infrared wave band to avoid significant light absorption in human tissue. Light‐absorbing agents are activated by exposure to light at a specific wavelength. The generated heat can then induce localized hyperthermia, which may result in the targeted destruction or damage of cells or tissues. To avoid light absorption in human tissue, the optical characteristics of light‐absorbing agents should be engineered to have high absorption in near‐infrared region (typical gold NPs have an absorption peak near 530 nm). For the reason, PTT using plasmonic heating adopted metallic nanoshells,^[^ 281, 282 ^]^ nanocages,^[^ ^283^ ^]^ NRs,^[^ ^284^ ^]^ and aggregated gold NPs.^[^ 285, 286 ^]^ Figure 13c shows spectral response of gold NRs with regard to aspect ratios and TEM images to be used in plasmonic PTT. Switching plasmonics described in this review have also been used to manipulate optical characteristics of plasmonic NPs for therapeutic applications. For example, Baffou and co‐workers used plasmonic heating to control heat‐shock response at a single cell.^[^ ^287^ ^]^ Bahadori et al.^[^ ^242^ ^]^ reported complete cell fusion of two HEK293 cells using heated gold NPs.

Note also that localized thermal distribution induced by plasmonic heating gives rise to an extreme temperature gradient which causes thermophoretic force to trap NPs and local convection flows in a liquid environment. Quidant and co‐workers reported plasmon‐assisted optofluidics with a gold disk as nano‐/microscale heat source and theoretically analyzed the coupling between light–heat–fluid in plasmonic nanostructures.^[^ ^244^ ^]^ In mid‐2010, Ho and co‐workers utilized thermophoretic forces that are induced by an extreme temperature gradient to trap, sort, and transfer nano‐/microparticles and live cells.^[^ 245, 246 ^]^ As shown in Figure 13d, thermophoretic force induced by plasmonic heating overcomes optical gradient force to trap and assemble *Escherichia coli* as well as polystyrene spheres on gold nanoisland substrate.^[^ ^245^ ^]^ These methods have a potential to manipulate nano‐/microsized objects which can be delivered to targeted microorganisms and be reacted for treatment.

### 4.3 Dynamic Optical Device Applications: Plasmonic Color Displays

Plasmonic color display is a fascinating technology that offers the potential for high‐resolution, vivid color imaging with nanoscale structures. Conventional plasmonic color generation is achieved by exploiting scattering or absorption of light by metal NPs or nanostructures, which can be precisely engineered to produce a wide range of colors.^[^ 288, 289 ^]^ To implement a dynamic color display, many groups have investigated various plasmonic switching techniques, which we describe in this section.

**Figure 14:**
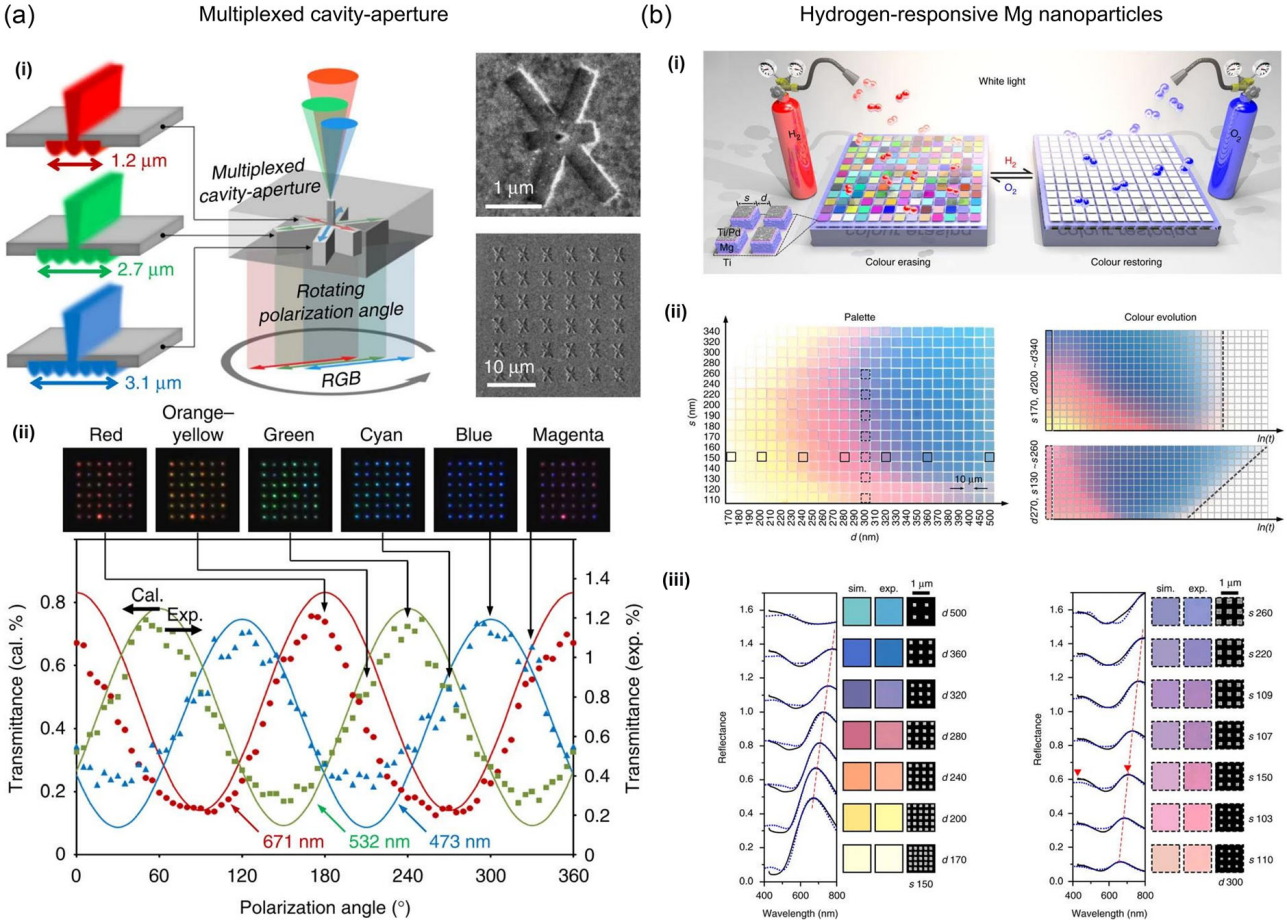
Plasmonic color display. a) Multiplexed cavity‐aperture‐based plasmonic color pixel. i) Schematic illustration of the multiplexed cavity aperture and its SEM images of a unit pixel and pixel array. ii) Transmittance images captured by optical microscopy of the multiplexed cavity aperture array to present colors versus polarization angle. The graph presents the absolute transmittance according to the polarization angle in both simulation and experimental results. a) Reproduced under the terms of the CC‐BY Creative Commons Attribution 4.0 International license (https://creativecommons.org/licenses/by/4.0).^[^ ^290^ ^]^ Copyright 2015, The Authors, published by Springer Nature. b) Catalytic magnesium metasurfaces based plasmonic color display. i) Schematic illustration of the plasmonic metasurface composed of magnesium NPs that are responsive to hydrogen, interacting with unpolarized incident white light. ii) Color palette obtained by incrementally tuning the s and d parameters (left), and the modifications in color of chosen color squares over time (ln(t)) upon exposure to hydrogen, with the gray dashed lines indicating the time at which the color disappears in both cases (right). iii) Experimental (black) and simulated (blue‐dotted) reflectance spectra of chosen color squares from the color palette before undergoing evolution. For clarity, the spectral curves have been shifted upward. b) Reproduced under the terms of the CC‐BY Creative Commons Attribution 4.0 International license (https://creativecommons.org/licenses/by/4.0).^[^ ^293^ ^]^ Copyright 2017, The Authors, published by Springer Nature.

First, passive plasmonic switching is one of the basic strategies for plasmonic color display. This approach requires plasmonic nanostructures to be well designed for convenient passive switching and light sources to be well controlled for fine tuning. Yun et al.^[^ ^290^ ^]^ reported a design that features metallic cavity and nanoaperture, which is termed cavity aperture, which allows the concurrent tuning of color and intensity of transmitted light within a single pixel. The metal cavity established plasmonic standing waves that regulate the spatial distribution of amplitudes based on wavelength, while the nanoaperture enables the transmission of light with a specific wavelength and amplitude, depending on the relative location within the cavity and the polarization state of incident light. As a result, the cavity aperture possessed the capacity to act as a versatile color pixel. **Figure** **14**a shows the device structure, SEM images of the pixel components, and demonstrates the operational performance of the dynamic plasmonic pixels. Various active plasmonic switching methods, such as chemical‐based switching, electrical switching, and combination of multiple switching approaches, were attempted for plasmonic color display.^[^ 291, 292 ^]^ Duan et al.^[^ ^293^ ^]^ reported plasmonic color display that is adaptable and changeable, which utilizes catalytic magnesium metasurfaces. Printing, tuning, erasing, and restoring of plasmonic color are facilitated by regulated hydrogenation and dehydrogenation using magnesium NPs as dynamic pixels. Each dynamic pixel possesses distinct color transformation kinetics, providing the ability of plasmonic animation. Figure 14b presents the working principle of a dynamic plasmonic color display, including a color palette and the evolution of color with hydrogen exposure over time, as well as simulation and experimental results of selected individual pixels.

**Figure 15:**
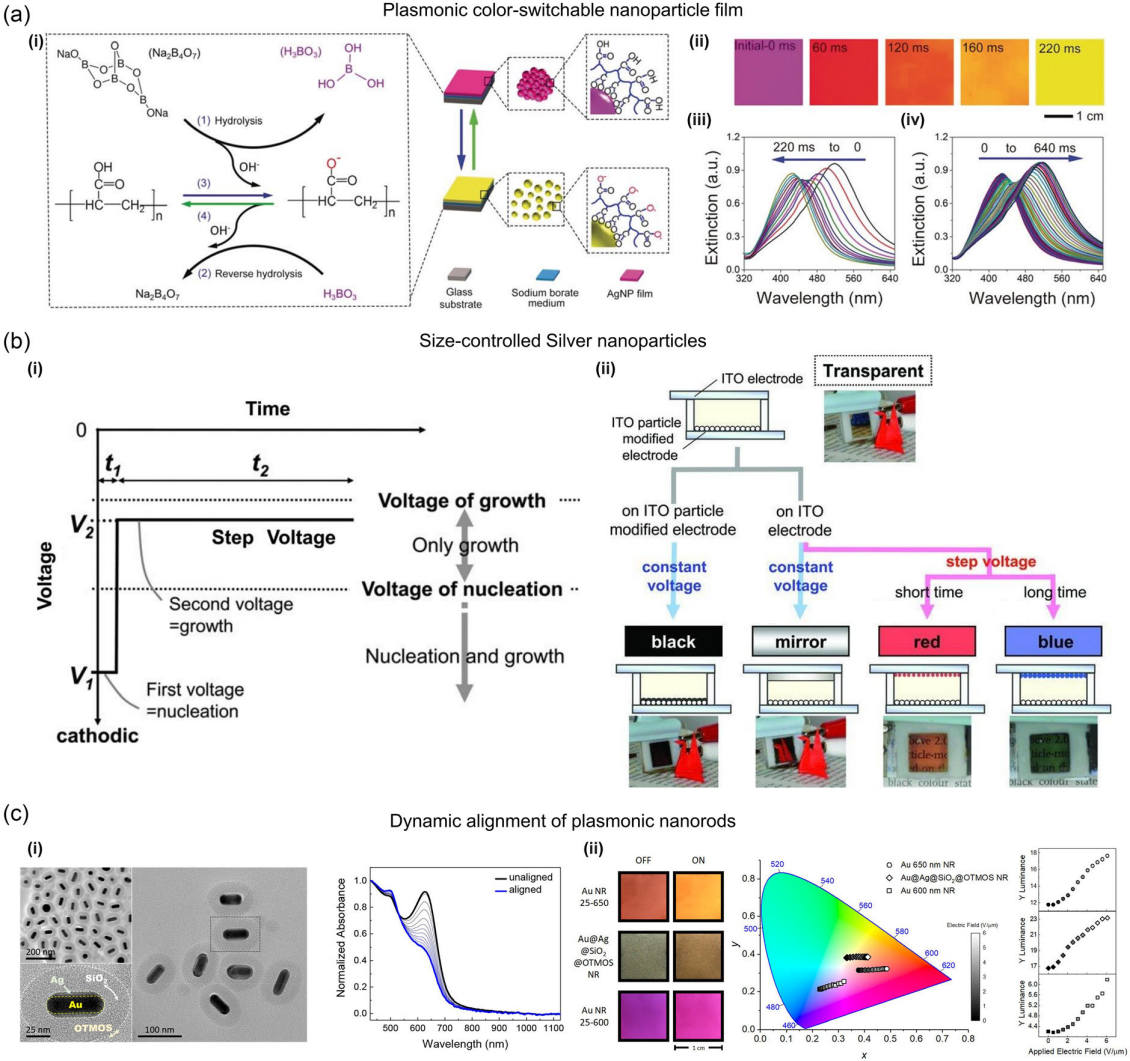
Chemical reaction‐based plasmonic color display. a) Plasmonic color‐switchable silver NP films. i) Schematic for design of a color‐switchable silver NP film. The left‐hand diagram depicts the chemical reactions that take place, while the right‐hand diagram illustrates the modification in the assembly structure of the silver NP film that occurs during the plasmonic color switching process. ii) Reversible plasmonic color switching of a conventional silver NP film. Photographs of the plasmonic color switching process, depicting the transformation from pink to red, orange, and ultimately yellow. iii,iv) Extinction spectra of the standard silver NP film, with: iii) presenting the spectra upon the application of moisture and iv) presenting the spectra upon its subsequent removal. a) Reproduced with permission.^[^ ^294^ ^]^ Copyright 2019, Wiley‐VCH. b) Voltage‐step method‐based dynamic plasmonic color switching. i) Schematic illustration of the voltage‐step method. ii) The present study provides a depiction and visual representation of a two‐electrode electrochromic cell. The cell exhibits distinct optical states, including a transparent state observed before voltage application, a black state evident during a constant voltage application of +2.5 V for 20 s, a mirror state observed during constant‐voltage application of −2.5 V for 20 s, a red state observable during step‐voltage application of −4.0 V for 20 ms and −1.6 V for 1 s, and a blue state evident during step‐voltage application of −4.0 V for 20 ms and −1.6 V for 3 s. b) Reproduced with permission.^[^ ^297^ ^]^ Copyright 2013, Wiley‐VCH. c) Dynamic plasmonic pixels that operate through the alignment of plasmonic NRs. i) TEM images of Au@Ag@SiO_2_@OTMOS (octadecyltrimethoxysilane, OTMOS) NRs in different magnifications (left). Experimental result of the extinction spectra of Au@Ag@SiO_2_@OTMOS NRs in heptane is presented according to the alignment state with varying external electric fields (right). ii) Color photographs of the indicated gold NR suspensions when an applied external electric field is either off or on (left). Plotting of chromaticity coordinates *x, y* based on the CIE 1931 color space (center), along with luminance values as a function of the applied electric field (right). c) Reproduced with permission.^[^ ^203^ ^]^ Copyright 2019, American Chemical Society.

Just as chemical–stimuli response is one of the popular ways to achieve plasmonic switching, chemical reaction‐based dynamic color generation has been widely investigated. Liu et al.^[^ ^294^ ^]^ reported an innovative approach for producing plasmonic color‐switchable silver NP films. They employed poly(acrylic acid) (PAA) as a capping ligand and sodium borate as a salt. Upon exposure to moisture, borate hydrolyzes rapidly and generates OH^−^ ions, which deprotonate PAA on silver NPs. This results in a change in the surface charge and facilitates the reversible adjustment of plasmonic coupling among adjacent silver NPs to produce plasmonic color switching. These plasmonic films can be printed as high‐resolution invisible patterns, which can be effortlessly uncovered with high contrast by exposing them to trace amounts of water vapor. **Figure** **15**a shows a schematic illustration of plasmonic color‐switchable silver NP film with its working principle and experimental results of optical properties that show the reversible performance of the plasmonic color switching. Chemical reactions are based on electronic events in the molecular regime; thus, there have been many strategies to implement dynamic color generation through electrochemical methods.^[^ 295, 296 ^]^ Tsuboi et al.^[^ ^297^ ^]^ reported the “voltage‐step method,” which employs a silver deposition‐based electrochromic device to achieve reversible changes in multiple colors by shifting the LSPR band. In this technique, two distinct voltages are administered consecutively (depicted in Figure 15b): the first voltage, *V*_1_, is applied briefly for a short time period, *t*_1_, to trigger silver nucleation. Subsequently, the second voltage, *V*_2_, is applied for a duration of time, *t*_2_, to encourage the growth of the silver nuclei. Because *V*_2_ is more positive than the nucleation voltage, nucleation ceases during *t*_2_. As a result, the growth of the silver particles and the resulting transmission spectra of the device can be modulated by altering *t*_2_. Figure 15b presents a schematic illustration with the experimental results that explain how a plasmonic multicolor electrochromic device works. Wang et al.^[^ ^298^ ^]^ described a technique for plasmonic modulation using bimetallic nanodot array and electrochemical bias. Their method offers a notable advantage of enabling real‐time light manipulation that can be adapted to the color characteristics of a specific environment. They employed this capability to produce a biomimetic mechanical chameleon and an active matrix display that are capable of dynamically rendering colors across almost the entire visible region.

Electric field‐based alignment of anisotropic plasmonic materials has been a popular method for switching plasmonic fields. Most groups to exploit this strategy have mainly adopted liquid crystals to implement modulation of the intensity of local plasmonic pixels like liquid crystal display^[^ 29, 299, 300, 301, 302, 303 ^]^ or modulate plasmonic materials themselves by attaching liquid crystals.^[^ 304, 305 ^]^ In this scenario, conventional plasmonic switching using liquid crystals is limited by the performance of liquid crystals, such as operating speed. Greybush et al.^[^ ^203^ ^]^ exhibited the ability to control light in terms of its spatial, spectral, and temporal characteristics using dynamic plasmonic pixels that operate through the alignment of plasmonic NRs in organic suspensions induced by an electric field. By manipulating the geometry and composition of the NRs (Au and Au@Ag), they demonstrated light modulation across a wide range of wavelengths in both the visible and infrared regions (600–2400 nm). The rapid (≈30 μs) and reversible alignment of the NRs caused perceptible changes in color, as indicated by shifts in the observed chromaticity and luminance. Figure 15c shows TEM images of plasmonic active particles and experimental results according to exerting external electric fields to align the particles, as well as the experimental results of coloring with dynamic plasmonic pixels.

## 5 Summary and Outlook

### 5.1 Switchable Plasmonic Nanostructures: A New Frontier

This section explores some recent developments in this rapidly evolving field of switchable plasmonic nanostructures. We first introduce the concept of inverse design, which enables the rapid and efficient optimization of plasmonic nanostructures with the desired optical properties. We then discuss hybrid plasmonic structures that combine different types of materials and geometries to achieve enhanced functionality. Finally, we explore the potential of quantum plasmonic structures that use quantum effects to control light–matter interactions in the quantum regime. This section provides a comprehensive overview of the emerging field of switchable plasmonic nanostructures and their potential for transforming various fields of science and technology.

#### 5.1.1 Inverse Design in Switching Plasmonics

Inverse design is an essential concept in plasmonics that attempts to control and manipulate plasmons to achieve highly efficient and tunable optical switching and modulation. An inverse design in switching plasmonics is necessary because of plasmonic systems’ complex and nonlinear nature,^[^ 306, 307 ^]^ with the behavior of plasmonic devices highly dependent on their geometry, material, and surrounding environment. In addition, small changes in these parameters significantly impact the performance. Gradient‐based algorithms and gradient‐free methods, along with neural nets and Bayesian optimization, are the two primary optimization techniques currently employed for reverse engineering plasmonic structures.^[^ ^308^ ^]^ By leveraging advanced computational algorithms and simulation tools, researchers can simulate the behavior of plasmonic structures and optimize their performance for various design parameters such as shape, size, material composition, and external stimuli. Many approaches, including evolutionary/genetic algorithms^[^ 309, 310, 311 ^]^ and topology optimization,^[^ 312, 313 ^]^ have been employed to design and optimize the performance of plasmonics. In addition, deep learning was also utilized with various techniques such as bidirectional/tandem neural network,^[^ 314, 315, 316 ^]^ iterative neural network,^[^ 317, 318 ^]^ generative neural network,^[^ 319, 320, 321, 322 ^]^ and dimension reduction.^[^ 323, 324 ^]^

These methods can be extended to plasmonic switching. Bayesian optimization was used to design the phase profile of the input electromagnetic wave to achieve the desired local optical field on the plasmonic metasurface.^[^ ^325^ ^]^ In this approach, a continuous phase profile, represented by six variables, was assumed to be incident on the I‐beam‐shaped plasmonic meta‐atoms. A metric was then defined to measure the relative field intensity ratio between the meta‐atoms. A figure of merit was established by selecting certain meta‐atoms with high localized field intensities, whereas others had low localized field intensities. The Bayesian optimization algorithm was employed as a global optimizer to maximize the figure of merit. The optimized phase of the illumination source resulted in on‐demand hotspot distributions by overcoming the optical coupling between neighboring meta‐atoms.

Genetic algorithms and neural networks were combined to manipulate the near‐field distribution of plasmonic nanoantennas.^[^ ^326^ ^]^ While genetic algorithms can generate diverse datasets of near‐optimal and pseudorandom solutions, generalizing the results is challenging because they require optimization for each unique sample and experimental arrangement. In contrast, pretrained neural networks can be easily adjusted and reused for similar problem sets, such as those with varying aspect ratios of plasmonic nanoantennas. By utilizing a shallow neural network in conjunction with genetic algorithms, optimization of switching the hotspot positions and maximization of absolute value of second harmonic flux was achieved.

#### 5.1.2 Hybrid Plasmonic Structures with 2D Materials

Compared with conventional bulk materials, 2D materials are atomically thick and exhibit unique electronic, optical, chemical, and mechanical properties.^[^ ^327^ ^]^ Owing to their electron confinement within two dimensions, 2D materials have high surface‐to‐volume ratios, leading to strong light–matter interactions and unique optical properties.^[^ 328, 329 ^]^ The properties of 2D materials can be tuned in situ using various methods such as electrical gating, strain stimuli, magnetic fields, and thermal heating to expand the functionalities of optoelectronic devices.^[^ ^330^ ^]^ For example, nanopatterned graphene can act as an active medium for infrared electro‐optic devices, with its enhanced absorption efficiency being voltage tunable.^[^ ^331^ ^]^ In addition, a graphene‐based broadband optical modulator was developed, in which the Fermi level of graphene was modulated by applying a drive voltage with the integration of the waveguide. This scheme exhibited strong electroabsorption modulation over a broad range of wavelengths.^[^ ^332^ ^]^ Apart from graphene, transition metal dichalcogenides possess electrically tunable optical properties, achieved by manipulating the Fermi level and exploiting the excitonic effect.^[^ 333, 334 ^]^ In contrast, electro‐optic modulation of black phosphorus was achieved by leveraging the quantum‐confined Franz–Keldysh effect.^[^ 335, 336 ^]^

Integrating 2D materials with metal structures has shown considerable potential in modulation and switching applications by overcoming weak light–matter interactions and low light‐harvesting efficiencies.^[^ 337, 338 ^]^ Plasmonic resonance was controlled in the near‐infrared and infrared regions using a hybrid gold structure with electrical gating.^[^ ^339^ ^]^ The Fano resonance was also electrically modulated with a graphene field‐effect transistor, which can be extended to the reflectivity modulation of light.^[^ 340, 341 ^]^ In addition, plasmons can be thermally controlled using graphene, which induces a stronger photothermal response.^[^ ^342^ ^]^

#### 5.1.3 Quantum Regime Applications

In the quantum regime, plasmons can exhibit properties different from those of their classical counterparts. Plasmon‐driven light–matter interactions can be modified by quantum effects such as quantum confinement, tunneling,^[^ ^343^ ^]^ and entanglement.^[^ ^344^ ^]^ In 2002, quantum entanglement was maintained when a polarization‐entangled pair of photons impinging on a gold grating were converted to SPP and back to photons.^[^ ^344^ ^]^ Since then, various experimental variations have been reported, such as energy–time‐entangled photons using a long‐range SPP waveguide,^[^ 345, 346 ^]^ entanglement of orbital angular momentum,^[^ ^347^ ^]^ polarization,^[^ ^348^ ^]^ and path entanglement.^[^ ^349^ ^]^ However, in most cases, the light–matter interaction between light and single emitters is weak, owing to the size mismatch between them. Several approaches have been proposed to overcome this weak coupling efficiency, including high‐quality cavities and extreme light confinement.^[^ 350, 351 ^]^ However, a high‐quality cavity may limit the bandwidth of the response and the device size. Therefore, in 2007, Akimov et al.^[^ ^351^ ^]^ generated a single SP using silver nanowires to excite the subwavelength confinement of optical near‐fields, with the results suggesting that nonclassical photon correlations between the nanowire and quantum dot are related to the generation of quantized plasmons. Considering this pioneering work related to single‐plasmon generation, several attempts to improve and control the coupling efficiency between single emitters and SP have been reported.^[^ 352, 353, 354, 355 ^]^ Moreover, metamaterials have been developed as one of the technologies to investigate light–matter interactions in the quantum regime.^[^ ^356^ ^]^ Switching plasmonic characteristics in the quantum regime opens up the possibility of new methodologies in quantum communication and quantum computing applications.

## 6 Conclusion

This review article provided an overview of dynamic plasmonic switching and engineered nanostructures and their principles, methodologies, and applications. Passive and active strategies are used to engineer switchable plasmonic nanostructures, enabling various applications in biomedical engineering, energy, and dynamic optical devices. Emerging trends in the engineering of switchable nanostructures were also discussed. Inverse design in switching plasmonics, which refers to using computational techniques to design plasmonic structures with the desired properties, was discussed to create new and more complex structures. Hybrid plasmonic structures with 2D materials are another emerging trend in switchable nanostructure engineering. By combining plasmonic structures with 2D materials such as graphene, researchers can achieve enhanced performance and unique functionalities such as strong light–matter interactions. In the quantum regime, plasmonic switching can control the quantum states of matter and light, enabling new applications in quantum communication and computing.

Some challenges must be addressed to fully realize the potential of plasmonic switching. One challenge is the scalability and reproducibility of fabrication processes because plasmonic nanostructures often require high‐resolution lithography and complex material synthesis. Another challenge is the stability and reliability of the switching mechanisms because external stimuli can cause irreversible damage or degradation of the nanostructure. In future research, we expect that various passive and active strategies for switching, as well as different materials for active switching, will provide a wide range of options for manipulating light at the nanoscale level with unprecedented tunability and versatility.

## Conflict of Interest

The authors declare no conflict of interest.
